# Short- and long-term effects of a need-supportive physical activity intervention among patients with type 2 diabetes mellitus: A randomized controlled pilot trial

**DOI:** 10.1371/journal.pone.0174805

**Published:** 2017-04-06

**Authors:** Jari Vanroy, Jan Seghers, An Bogaerts, Karlien Devloo, Stijn De Cock, Filip Boen

**Affiliations:** 1 Department of Kinesiology, KU Leuven, Leuven, Flemish Brabant, Belgium; 2 Faculty of Kinesiology and Rehabilitation Sciences, KU Leuven, Leuven, Flemish Brabant, Belgium; 3 Department of Health Promotion, Christian Health Insurance Fund, Brussels, Flemish Brabant, Belgium; Florida International University Herbert Wertheim College of Medicine, UNITED STATES

## Abstract

**Objective:**

This pilot trial evaluated the short- and long-term effects of a six-week need-supportive physical activity (PA) intervention among patients with type 2 diabetes mellitus, on health-related (HbA1c and physical fitness) and behavioral (objectively-measured and self-reported PA) outcomes.

**Methods:**

To support the basic psychological needs for autonomy, relatedness and competence, the intervention included one in- and outtake session with a PA coach, an individualized PA program and a weekly PA group session. The intervention was set up in collaboration with a health insurance fund and with general practitioners. A total of forty-eight patients participated in the study and were randomly assigned to an intervention (*n* = 27) or a waiting-list control condition (*n* = 21).

**Results:**

Linear mixed models did not reveal any significant interaction effects between time and condition (*p*s > .05). However, significant time effects across conditions were found: a decrease in HbA1c at short term and increases in self-reported PA at both short and long term and in physical fitness at long term (*p*s < .05).

**Conclusion:**

Although the intervention as a whole did not produce the expected impact, there seems a potential for brief but regular expert visit and measurement.

## Introduction

The World Health Organization (WHO) projects that diabetes will be the seventh leading cause of death in 2030 [1]. The most common type of this rising disease is type 2 diabetes mellitus (T2DM) [2]. Diabetes reduces not only life-expectancy but also life–quality. However, it has become largely manageable due to advances in medication and diet. In addition to these traditional methods, a third viable strategy has received increasing attention, namely physical activity (PA). In this regard, a systematic review suggested that different types of PA interventions can contribute to (potentially clinically relevant) reductions in blood sugar level (HbA1c) of about 0.5 percentage points [3]. The duration of the interventions included in this review was rather long (at least twelve weeks) and some of these interventions comprised a dietary co-intervention. Nonetheless, studies in the area of diabetes management have rarely provided a(n) (PA) intervention with conclusive, translatable results [4, 5]. Based on these studies, the current pilot study applied a randomized controlled field trial with a six week PA intervention to address four common pitfalls.

First, in the PA intervention domain, in relation to diabetes management and to health promotion in general, a trend is emerging to elicit sustained behavior change through an amalgam of techniques, whether based on psychological theories or not (e.g., [6]). This approach makes it difficult to obtain an overarching communication structure [7], let alone theoretical coherence. Therefore, in the current intervention, the Self-Determination Theory (SDT) was used as a theoretical framework [8]. This framework offers a clear vocabulary with principled touchstones as well as possible means to meet these principles. The theory stems from an organismic view of man, thereby focusing on humans’ potential for growth under circumstances that satisfy three innate, essential and universal basic psychological needs: autonomy–to act volitional (with choice, self-authored), relatedness–to belong to a caring relationship-structure, and competence–to master the environment. These three needs were supported in the current intervention. Several studies have shown sustainable effects of SDT-based interventions on PA behavior, also in clinical populations [9]. Additionally, it has been shown that changes in HbA1c over time can be predicted by SDT-derived variables [10].

Second, many recruitment and screening procedures have prevented participation of people who could benefit the most from (PA) interventions, for example those from minority groups or those with comorbid chronic illnesses [4]. Such people might be reached through their health insurance fund, at least in Belgium, where health insurance is obligatory. Therefore, in the current intervention, staff members from the Christian Health Insurance Fund of Leuven (CM Leuven) took care of the recruitments. They used a novel method, which included personal writings based on a database of patients with T2DM, information sessions and e-mails.

Third, participants’ general practitioner (GP) was involved in the current intervention. This approach has been applied in previous interventions only to a limited extend but seems promising, provided that the extra workload for GPs remains easy to implement [4]. In a similar vein, a study showed small effects of a GP-intervention (prescription and written materials) on self-reported PA among inactive routine care patients at short term (6–10 weeks) [11].

Fourth, most studies in patients with T2DM that measured regular PA in an objective way, relied on either pedometry or accelerometry [12]. Therefore, in the current pilot study, a multisensory activity monitor (Sensewear) was used to measure regular PA. This device takes physiological measures (e.g., heat flux) into account as well as mechanical ones (e.g., accelerations). In addition, a self-report questionnaire was used for triangulation.

We hypothesized that participants in an intervention condition (IC) would improve significantly more than participants in a waiting-list control condition (CC) from baseline to six weeks (intervention duration) and to six months follow-up, on blood sugar level (larger decrease in IC), physical fitness (larger increase in IC) (i.e., health-related outcomes), and on the behavioral outcome of regular PA (larger increase in IC). These outcomes were selected in line with the underlying study rationale of patients with T2DM improving their health through PA. The results from the current pilot study can inform research and health insurance funds regarding large scale rollout.

## Methods

### Design

The current pilot study consisted of a randomized controlled field trial. We used a 2 x 3 repeated measures design with condition (intervention vs. control) as between-persons variable and time (pre–post and pre–follow-up) as a within-persons variable. The two between-persons conditions were: an intervention condition (IC), in which participants were measured and received a 6-week intervention immediately after the baseline measurement; and a control (waiting) condition (CC), in which participants were measured during the study, as the IC, but would receive the intervention only after the follow-up measurement. Participants in the CC were told that during the waiting period their health measurements were analyzed. All participants had to pay a fee because CM aimed to initiate an economically feasible project that could be evaluated on its public health impact.

The measurement points were scheduled at three moments: at baseline, that is in spring 2014 (pre), six weeks after the baseline measurement, when the intervention had ended (post), and six months after the baseline measurement (follow-up).

### Participants

The inclusion criteria for participants were: (1) ≥ 18 years of age, (2) member of CM Leuven, (3) intake of oral diabetes medication (≥ three months since 2012), (4) no intake of Byetta/Victoza/insulin medication since 2012 (to exclude patients with type 1 diabetes mellitus), (5) in possession of a Global Medical File, which can be accessed by GPs and contains a medical history (e.g., examinations, medication, specialized care), and (6) resident within a region of about 40 km around Leuven.

Based on these criteria, a CM-database yielded 4273 eligible patients. After randomization (per GP, to avoid contamination between conditions), CM sent an invitation letter for an information session to 2310 patients in the IC and to 1963 in the CC. These information sessions were organized at four locations within the region and were identical in both conditions, except for the project schedule.

Of all invitees, 146 patients in the IC (6%) and 125 patients in the CC (6%) were present at one of the information sessions. Of these patients, thirty-six eventually subscribed for participation (26 IC, 10 CC). Two patients who were not present at the information session subscribed on their own initiative, based on the invitation letter (1 IC, 1 CC).

Because of the low participation rate in the CC after the information sessions, a sample of patients that had not subscribed for the information session were contacted through e-mail and/or telephone. This resulted in ten additional participants. In sum, twenty-seven participants subscribed for the IC, twenty-one for the CC.

**Fig 1:**
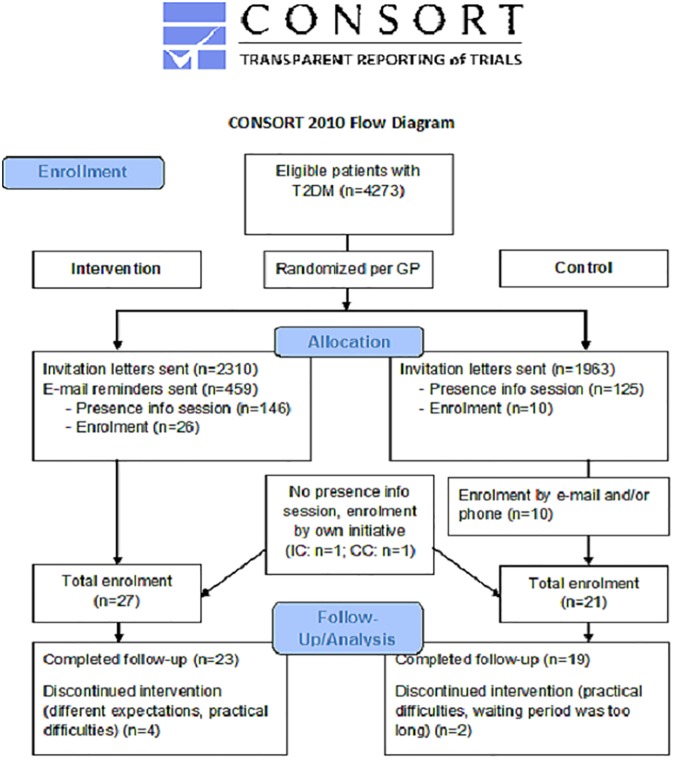
CONSORT flow chart.

All forty-eight participants (27 IC, 21 CC) signed an informed consent form and received medical approval by their GP. Thirty-six participants (20 IC, 16 CC) paid the full fee of €124.9, nine participants (5 IC, 4 CC) paid a reduced rate of €74.9 because of their low income, and three participants (2 IC, 1 CC) ended their participation prematurely before payment. The reasons for this premature cessation were practical difficulties (in two occasions) and/or different expectations (in two occasions). Of the forty-five remaining participants, forty-two participants completed the study, twenty-three in the IC and nineteen in the CC. The reasons for drop-out were practical difficulties (*n* = 1) or different expectations (*n* = 1) in the IC, or a waiting period that was too long (*n* = 1) in the CC. Fig 1 summarizes the recruitment process flow. This study (ML9981) was approved by the Medical Ethics Committee of the University Hospitals Leuven (ClinicalTrialsID: NCT02064335).

### General practitioners

All eligible GPs were informed about the project by CM through a standardized information letter. They were asked: to screen patients that were interested to participate, to fill out a brief questionnaire with questions about the project quality and feasibility (if they had a participating patient), to list information about the medication of participating patients, to collect blood sugar and cholesterol levels of participating patients and to discuss these levels with them. These levels were based on blood samples incorporated into the three-monthly routine investigations.

### Intervention

The current SDT-based PA intervention focused on the satisfaction of participants’ basic psychological needs (i.e., autonomy, relatedness and competence) and consisted of three key elements: (1) an intake and an outtake session with a professional PA coach, who held the degree of Master in Physical Education and Movement Sciences and who was familiarized with SDT and Motivational Interviewing [13], (2) a personalized PA program and (3) five weekly PA group sessions.

The intake and outtake session included measurements, discussions about personal scores for the measured variables of interest (e.g., blood sugar) and the composition of a personalized PA program. The intake session had been preceded by an acquaintance session, one week earlier. In this acquaintance session, the multisensory armband was handed because this device has to be worn for a week to obtain a valid (pre) measurement.

The personalized PA program was drafted through dialogue between participant and PA coach, in line with psychological principles from SDT and related practices from Motivational Interviewing [13]. Physiological training principles were taken into account too: duration, frequency and intensity ranges were adapted to the individual fitness level. The type of PA could be easily integrated into participants’ daily life. Walking and cycling were the most selected personal activities. The participants also received self-monitor tools such as a pedometer and a booklet that included a PA diary as well as practical and health-related information.

The weekly group sessions were guided by the PA coach and consisted of either walking (*n* = 10), Nordic Walking (i.e., walking with poles) (*n* = 13) or aerobic and strength training (*n* = 2). This type of activity was free to choose for participants. Each session lasted for about one hour and took place at one of the four locations from the information sessions. Based on participants’ location and PA preference, groups were composed, in which the number of participants varied from one to five. All but two participants completed all five PA group sessions. Table 1 shows how the three key intervention elements are related to the three dimensions of need support, in accordance with SDT [14].

**Table 1:** Links between key intervention elements and need support dimensions.

	Autonomy	Relatedness	Competence
Intake & outtake	• MI to consider choice • discuss past experience & expected difficulties	• support through planning, agreeing & reviewing PA • support from PA coach to identify with PA	• understand PA • consider PA types • pros/cons PA • success/failure reflection • value/competence beliefs
PA program	• self-regulation and PA ownership	• information provision	• small achievable steps • progress monitoring • self-regulatory skills • mastery focus
Group sessions		• support from significant others • attachments with others through PA • self-perception in social settings	

### Measurements

#### Demographics

Demographic sample characteristics were determined by means of a questionnaire and are given in Table 2 for the baseline measurement. The IC and CC differed significantly at baseline with respect to age (Mann-Whitney U: *p* = 0.012) and work status (2-sided Fisher’s Exact: *p* = 0.021). These results mean that the IC contained a higher proportion of retired (older) people.

**Table 2:** Demographic baseline characteristics.

	IC (*n* = 25)	CC (*n* = 21)
Age (*M*±*SD* years)	65.3±8.1^a^	59.4±8.2
Sex (*n* males)	17^a^	10
Relation (*n* people in a relationship)	19	18
Work (*n* retired people)	16	6
Education (*n* higher educated people)	10	10
Parent (*n* people with ≥ 1 child)	19	15

^a.^ Based on a sample size of 27 participants.

#### Health-related outcomes

HbA1c was used as a measure of blood sugar level and determined by means of the blood sample, taken by participants’ GP. Values expressed in mole/mole (IFCC- HbA1c) were converted to a percentage (NGSP- HbA1c), according to the formula: *NGSP-HbA1c = 0*.*915 x (IFCC-HbA1c) x 100% + 2*.*15%* [15].

Distance walked in six minutes was used as a measure of physical fitness and determined by means of the six-minute walk test, guided by the PA coach. In this test, participants had to walk as many 20m-lenghts as possible within six minutes [16]. Physical fitness is considered relevant to health in its own way [17] and has been shown to be amendable through PA interventions [18].

#### Behavioral outcome

Regular PA was assessed in an objective way by means of a validated multisensory armband (SenseWear) [19]. The armband had to be worn at the upper arm continuously for one week, except during water activities. A minimum of three valid weekdays and one valid weekend day was considered a valid measurement week [20, 21]. A minimum of twelve awake wearing hours was considered a valid day [22]. The output consisted of the weighted mean daily amount (*5/7 x weekday mean + 2/7 x weekend-day mean*) of time spent on PA at intensity above 1.8 MET, in bouts of at least ten minutes. Because of very low scores on vigorous PA in our sample (at baseline: overall *M* < 1 min/day), we did not differentiate between PA intensity levels.

Regular PA was assessed in a subjective way too by means of an adapted version of a self-report questionnaire (Godin Leisure-Time Exercise Questionnaire; [23), to be completed at home. Participants had to report how many times during the previous week they had been engaging in light (L), moderate (M) and vigorous (V) PA in bouts of at least fifteen minutes. A total (T) PA-score was calculated according to the formula: *TPA = 3 x LPA + 5 x MPA + 9 x VPA* [23]. Within the current study, this total PA-score seemed the most appropriate subjective measure of variation in PA behavior because it could be compared with the objective PA-score and because walking (often classified as light PA) was a popular activity among participants.

#### Covariates

Age, sex, BMI, climatic circumstances and dietary changes were assessed as covariates. To determine BMI (i.e., body mass/height²), body mass was measured by means of a digital balance (OMRON) and height by means of a stadiometer. On an interpersonal level, BMI can be used as a feasible proximal measure to detect people with overweight, which might hamper PA behavior [24].

Three categories of the climatic circumstances during the week of PA-assessment (objectively) were determined: mean day length (in Brussels), based on data from the Royal Observatory of Belgium (ROM), mean day temperature and total day precipitation (both in Leuven), the last two based on data from the Royal Meteorological Institute of Belgium (RMI). It has been shown that climatic circumstances play a role in people’s PA behavior [25].

Two categories of dietary changes compared with the baseline measurement were determined: sugar intake and total energy intake, both measured by means of a question to participants with a trichotomous answer possibility (less/equal/more). Because of the ordinal nature of the data and because the intervention did not target diet, these variables were not measured as an outcome in themselves.

Based on self-report, there were six smokers at baseline (*n* = 45). Only four of them changed their amount of smoking during the study, and only to a minimal extent (< 2 cigarettes/day). Therefore, smoking information was not included as a covariate.

### Analyses

All statistical analyses were performed by use of SPSS 23. The significance criterion was set two-sided, at *p* < 0.05. No correction for multiple testing was used. In order to compare progress over time (assumed linear), we composed linear mixed models with condition (and the covariates) and time (measurement point) as (repeated, in case of time) fixed factors. We opted for unstructured covariance matrices and restricted maximum likelihood estimations.

## Results

### Sample

All twenty-three remaining participants in the IC completed the intake and outtake session with the PA coach and the five group sessions. Shapiro-Wilk tests indicated in general that data on HbA1c and self-reported PA were skewed. Therefore, these data were transformed into their logarithm (after addition of 1), which yielded satisfactory improvements in symmetry.

### Short-term effects

From pre to post, the hypothesized interaction effects (condition x time) were not significant, which denotes a lack of difference in progress on the outcome variables between both conditions. However, significant (six-week) time effects across conditions were found for blood sugar levels and for self-reported PA. More specifically, the HbA1c levels decreased significantly (on average: -0.19%), while the self-reported PA-scores increased significantly (on average: +6.46 points). These findings were identical for the model without (Table 3) and with (Table 4) covariates.

**Table 3:** Short-term effects–raw.

	Condition	Pre	Post	*F*	*F*	*F*
		(*M*±*SD*)^a^	(*M*±*SD*)^a^	condition	time	condition x time
						x time
HbA1c	IC	6.7	6.5	0.13	10.13*	0.63
(%)	CC	6.4	6.4			
Physical fitness	IC	521±83	530±97	0.26	3.23	0.08
(m)	CC	539±88	545±85			
PA–objectively	IC	144±81	148±78	0.21	0.12	0.20
(minutes/day)	CC	129±59	123±76			
PA–subjectively	IC	21.5	26	0.04	6.17*	0.14
(score)	CC	33.5	33.5			

^a.^ For HbA1c and self-reported PA, the median of the untransformed data is given.

*P < 0.05.

Note: sample sizes for the four outcome variables at both measurement points ranged between twenty-two and sixteen per condition. With regard to self-reported PA, two outliers (*> Q3 + 2*.*2 x interquartile range* or *< Q1–2*.*2 x interquartile range*) had been excluded. Other missing data were mainly due to practical issues such as incorrect use of the multisensory armband.

**Table 4:** Short-term effects–with covariates.

	Condition	Pre	Post	*F*	*F*	*F*
		(*M*±*SD*)^a^	(*M*±*SD*)^a^	condition	time	condition x time
						x time
HbA1c^b^	IC	6.7	6.5	0.19	8.54*	0.99
(%)	CC	6.4	6.4			
Physical fitness^c^	IC	521±83	530±97	0.00	2.91	0.81
(m)	CC	539±88	545±85			
PA–objectively^d^	IC	144±81	148±78	0.09	0.11	0.44
(minutes/day)	CC	129±59	123±76			
PA–subjectively^d^	IC	21.5	26	0.17	4.50*	0.53
(score)	CC	33.5	33.5			

^a.^ For HbA1c and self-reported PA, the median of the untransformed data is given.

^b.^ The following covariates were used: (1) age, (2) sex, change in (3) sugar intake and in (4) total energy intake.

^c.^ The following covariates were used: (1) age, (2) sex, (3) baseline BMI and (4) change in BMI (from pre to post).

^d.^ The following covariates were used: (1) age, (2) sex, (3) baseline BMI, (4) change in BMI (from pre to post), (5) day length (post), (6) temperature (post) and (7) precipitation (post).

*P < 0.05.

Note: ibid. Table 3.

### Long-term effects

From pre to follow-up, the hypothesized interaction effects (condition x time) were not significant. However, significant (six-month) time effects across conditions were found for physical fitness and for self-reported PA. More specifically, the distances walked in six minutes increased significantly (on average: +10.74m), as did the self-reported PA-scores (on average: +11.70 points). These findings were identical for the model without (Table 5) and with (Table 6) covariates.

**Table 5:** Long-term effects–raw.

	Condition	Pre	Post	*F*	*F*	*F*
		(*M*±*SD*)^a^	(*M*±*SD*)^a^	condition	time	condition x time
						x time
HbA1c	IC	6.9	6.6	0.06	0.56	0.53
(%)	CC	6.4	6.4			
Physical fitness	IC	525±83	541±90	0.12	5.39*	1.74
(m)	CC	541±91	545±82			
PA–objectively	IC	138±85	130±71	0.28	0.09	0.03
(minutes/day)	CC	131±64	120±71			
PA–subjectively	IC	22	36	0.00	4.23*	0.32
(score)	CC	33.5	31.5			

^a.^ For HbA1c and self-reported PA, the median of the untransformed data is given.

*P < 0.05.

Note: sample sizes for the four outcome variables at both measurement points ranged between twenty-three and sixteen per condition, except for the objective PA data in the CC (*n* = 12). Missing data were mainly due to practical issues such as incorrect use of the multisensory armband.

**Table 6:** Long-term effects–with covariates.

	Condition	Pre	Post	*F*	*F*	*F*
		(*M*±*SD*)^a^	(*M*±*SD*)^a^	condition	time	condition x time
						x time
HbA1c^b^	IC	6.9	6.6	0.09	1.48	0.43
(%)	CC	6.4	6.4			
Physical fitness^c^	IC	525±83	541±90	0.00	5.40*	1.78
(m)	CC	541±91	545±82			
PA–objectively^d^	IC	138±85	130±71	0.70	0.53	0.22
(minutes/day)	CC	131±64	120±71			
PA–subjectively^d^	IC	22	36	1.00	5.66*	1.26
(score)	CC	33.5	31.5			

^a.^ For HbA1c and self-reported PA, the median of the untransformed data is given.

^b.^ The following covariates were used: (1) age, (2) sex, change in (3) sugar intake and in (4) total energy intake.

^c.^ The following covariates were used: (1) age, (2) sex, (3) baseline BMI and (4) change in BMI (from pre to follow-up).

^d.^ The following covariates were used: (1) age, (2) sex, (3) baseline BMI, (4) change in BMI (from pre to follow-up), (5) day length (follow-up), (6) temperature (follow-up) and (7) precipitation (follow-up).

*P < 0.05.

Note: ibid. Table 5.

## Discussion and conclusion

### Discussion

The current pilot study evaluated the effects of a six-week need-supportive PA intervention on health-related and behavioral outcomes among patients with T2DM at short term (six weeks) and at long term (six months). The intervention took place in a real-life setting and was based on the principles of SDT. Positive and sustained effects were expected on blood sugar levels, fitness levels and regular PA.

In contrast to the hypotheses, no significant condition by time effects emerged at short or long term. This suggests that the intervention as a whole did not produce the expected impact. This finding is in line with the results of a recently conducted randomized controlled trial that aimed at the reduction of sedentary behavior among young adults at risk of T2DM [26]. That study did not show any significant intervention effects on HbA1c levels or PA behavior, nor at three, nor at twelve months.

However, three notable time effects across the two conditions were established in the current study. In a review of PA interventions, the authors concluded that eight of 29 (28%) studies reviewed reported meaningful improvements in PA behavior of participants in a control condition [27]. They suggested that repeated measurement and participant characteristics, such as a lower BMI and at risk for chronic disease (as opposed to healthy as well as to with chronic disease), were likely explanatory factors.

In the current study, a first time effect was indicated by a significant decrease in blood sugar level across the conditions after six weeks. This short-term effect is unlikely due to test familiarization, as HbA1c levels are based on blood samples, taken by a GP. Moreover, this effect is probably not caused by biased missing data either, as data on both HbA1c levels were available for forty participants. A possible explanation for the time effect lies in participants’ temporary focus on healthy living (related to blood sugar level), created by study enrollment. Participants might have become more aware of a variety of healthy behaviors, including PA but also sedentary time, medication adherence, diet etc., all of which might have influenced their blood sugar level. It is important to notice that this focus was not restricted to the IC; even though information and explicit need supportive counseling in the CC were minimized, it was not possible to ignore questions from patients in the CC altogether, nor was it desirable to thwart their basic psychological needs. Moreover, twenty-six patients in the IC and ten patients in the CC were recruited through an information session, which included PA- and health-related advice. Furthermore, the mere testing can provide informational feedback for self-monitoring. This feedback, in combination with the general advice and the contact with the PA coach (e.g., for measurement purposes), might have satisfied these patients’ basic psychological needs to a sufficient extent to increase their functional goal-directed health behavior.

A second time effect of the current study was indicated by significant increases in self-reported PA across conditions, both at short term as well as at long term. These effects are also unlikely caused by biased missing data, considering the sample size preservation rates of thirty-eight (83%) and thirty-nine (85%) participants respectively. Given that climatic circumstances were included as covariates, seasonal effects probably do not explain these effects either. Moreover, the baseline measurement took place in spring [28] and the total period between the first and the last measurement of all participants covered a wide range. Furthermore, about half of the participants were retired, which diminishes the probability of work-related variations.

In line with the time effect on blood sugar level, the focus on PA elicited by participation in the study might explain the time effect on self-reported PA. As stated above, a raised awareness could have elicited more goal-directed behavior towards PA both in the intervention condition as well as in the control condition. In addition, this raised awareness could have elicited increased recall of physical activities, independent of behavioral change. This increased recall might account for the discrepancy between self-reported and objectively measured PA. A cross-sectional study concluded that fitter participants showed larger discrepancies between self-reported and objectively measured PA than lesser fit ones because they tended to over-report more [29].

Another possible explanation for this discrepancy lies in the absence of differentiation in intensity levels in objectively measured PA, whereas the total PA-score based on self-report did account for differences in intensities. We opted not to differentiate between intensities in objectively measured PA in order to create a comprehensive and relevant measure of regular PA, with sufficient symmetry and variation in data. In addition, because of the ten-minute bouts, PA minutes from different intensity categories were not simply additive, which would complicate comparison. Moreover, objective data showed that ten-minute bouts of vigorous physical activities were quite rare (in contrast with the self-reported data), according to the cut-off value of 6 MET. When objectively measured light and moderate PA were considered separately in post hoc analyses, Wilcoxon Signed-Rank tests did not reveal any significant differences across conditions between pre versus post measurements (short term), nor between pre versus follow-up measurements (long term). Similar findings were obtained when light and moderate PA were analyzed without ten-minute bouts. Mann-Whitney U tests revealed only one notable significant difference, namely with respect to the long-term difference scores between the IC and the CC for objectively measured moderate PA without ten-minute bout restrictions. More specifically, the median difference score was positive in the IC whereas this difference score was negative in the CC. This finding suggests a positive long-term intervention effect on short periods of objectively measured moderate PA.

As a third and final time effect in the current study, the level of physical fitness increased significantly across the conditions at long term. If patients across both conditions indeed increased their regular PA, a likely consequence would be that there fitness levels also improved. This explanation seems especially appealing given the fact that walking was one of the most popular activities (at least in the IC) and that physical fitness was measured by a walking test. The lack of a time effect on physical fitness at short term might suggest a physiological adaptation period to the activity stimuli. It should be noted however that a positive trend was already noticeable at short term (*p* < 0.1).

The current pilot trial included several strengths. First, the study was ecologically valid because it took place in a real-life setting, in collaboration with patients’ health insurance fund. Second, GPs were involved but not burdened with heavy additional work-load. Third, the intervention was founded on one theoretical framework (SDT), which had been proven to be useful in different contexts. Fourth, several possibly important but less frequently handled covariates (e.g., climatic circumstances) were included. Fifth, PA was measured both objectively by means of a multisensory armband as well as subjectively by means of self-report.

The current pilot trial included a number of limitations as well. First, the total sample size was rather small. However, a similar study showed by means of power analysis (power = 0.80; *α* = 0.05) that a sample size of twenty patients in each condition should suffice to detect significant effects on regular PA (as measured by the number of steps per day) [6]. Moreover, to detect a clinically relevant change in HbA1C of 0.5% [30] at the within-subject level (i.e., across conditions), a sample size of thirteen participants seemed sufficient (power = 0.80; two-sided *α* = 0.05; paired *t*-test; *SD* = 1.0% [31]; *r*_within_ = 0.80) [32].

Second, the multisensory armband (Sensewear) had a few downsides. The device caused practical difficulties and loss of data and might have influenced PA behaviors. Moreover, the validity of this tool during walking has been questioned [19].

Third, no information about medication was taken into account. It should be noted though that during this study GPs only rarely reported changes in medication of participating patients. Nevertheless, future research should investigate changes in medication both as a covariate and as a valuable outcome in itself (i.e., independent of changes in blood sugar level). However, the willingness to experiment with medication taps into a cultural issue, which needs to be addressed but falls beyond the scope of this paper.

Fourth, the results cannot simply be generalized to the entire population of patients with T2DM. The fee in particular might have deterred potential participants. As a result, our sample is likely to be more motivated and/or economically advantaged compared with the non-participants.

### Conclusion

The PA intervention as a whole did not produce the expected effects in patients with T2DM. However, time effects suggest that certain aspects of the current project are effective. It would be of particular interest if these aspects could be proven to be not only effective but also relatively inexpensive. Hence, this pilot study suggests a potential for brief but regular expert visit and measurement, which could be incorporated into communal information sessions and short meetings with PA coach and GP.

## Supporting information

S1 Checklist CONSORT checklist. (PDF)

S1 File Dataset. (SAV)

S1 Protocol Dutch protocol. Note: this protocol was approved by the Medical Ethics Committee of the University Hospitals Leuven before the start of the trial.
(PDF)

S2 Protocol Translated protocol. (PDF)

S1 Supporting information Short-term effects. SPSS full models and output.
(DOC)

S2 Supporting information Long-term effects. SPSS full models and output.
(DOC)

## References

[1] Mathers CD, Loncar D. Projections of global mortality and burden of disease from 2002 to 2030. PLoS Med. 2006;3(11). 10.1371/journal.pmed.0030442 PMC1664601 17132052

[2] World Health Organization [Internet]. 10 facts about diabetes 2015 [updated 2016 April; cited 2016 Jul 15]. Available from: http://www.who.int/features/factfiles/diabetes/en/.

[3] Umpierre D, Ribeiro PAB, Kramer CK, Leitão CB, Zucatti ATN, Azevedo MJ, et al Physical activity advice only or structured exercise training and association with HbA1c levels in type 2 diabetes. A systematic review and meta-analysis. JAMA-J Am Med Assoc. 2011;305(17):1790–9. 10.1001/jama.2011.576 21540423

[4] Glasgow RE. Translating research to practice. Lessons learned, areas for improvement, and future directions. Diabetes Care. 2003;26(8):2451–6. 12882877 10.2337/diacare.26.8.2451

[5] Harris SB, Petrella RJ, Leadbetter W. Lifestyle interventions for type 2 diabetes. Relevance for clinical practice. Can Fam Physician. 2003;49(12):1618–25. 14708927 PMC2214163

[6] De Greef K, Deforche B, Tudor-Locke CE, De Bourdeaudhuij I. A cognitive-behavioural pedometer-based group intervention on physical activity and sedentary behaviour in individuals with type 2 diabetes. Health Educ Res. 2010;25(5):724–36. doi: 10.1093/her/cyq017 20338978 10.1093/her/cyq017 PMC2936553

[7] Michie S, Ashford S, Sniehotta FF, Dombrowski SU, Bishop A, French DP. A refined taxonomy of behaviour change techniques to help people change their physical activity and healthy eating behaviours: the CALO-RE taxonomy. Psychol Health. 2011;26(11):1479–98. doi: 10.1080/08870446.2010.540664 21678185 10.1080/08870446.2010.540664

[8] Deci EL, Ryan RM. Intrinsic motivation and self-determination in human behaviour New York: Plenum; 1985.

[9] Teixeira PJ, Carraça EV, Markland D, Silva MN, Ryan RM. Exercise, physical activity, and self-determination theory: A systematic review. Int J Behav Nutr Phy 2012;9(78). 10.1186/1479-5868-9-78 PMC3441783 22726453

[10] Williams GC, Freedman ZR, Deci EL. Supporting autonomy to motivate glucose control in patients with diabetes. Diabetes Care. 1998;21(10):1644–51. 9773724 10.2337/diacare.21.10.1644

[11] Smith BJ, Bauman AE, Bull FC, Booth ML, Harris MF. Promoting physical activity in general practice: a controlled trial of written advice and information materials. Br J Sports Med. 2000;34:262–7. doi: 10.1136/bjsm.34.4.262 10953898 10.1136/bjsm.34.4.262 PMC1724212

[12] Avery L, Flynn D, van Wersch A, Sniehotta FF, Trenell MI. Changing physical activity behavior in type 2 diabetes. A systematic review and meta-analysis of behavioral interventions. Diabetes Care. 2012;35(12):2681–9. doi: 10.2337/dc11-2452 23173137 10.2337/dc11-2452 PMC3507564

[13] Miller WR, Rollnick S. Motivational interviewing: preparing people to change addictive behavior. New York: Guilford Press; 1991.

[14] Haase AM, Taylor AH, Fox KR, Thorp H, Lewis G. Rationale and development of the physical activity counselling intervention for a pragmatic TRial of Exercise and Depression in the UK (TREAD-UK). Ment Health Phys Act. 2010;3(2): 85–91.

[15] Hoelzel W, Weykamp C, Jeppsson J-O, Miedema K, Barr JR, Goodall I, et al IFCC reference system for measurement of hemoglobin A1c in human blood and the national standardization schemes in the United States, Japan, and Sweden: A method-comparison study. Clin Chem. 2004;50(1):166–74. doi: 10.1373/clinchem.2003.024802 14709644 10.1373/clinchem.2003.024802

[16] Butland RJA, Pang J, Gross ER, Woodcock AA, Geddes DM. Two-, six-, and 12-minute walking tests in respiratory disease. Brit Med J 1982;284:1607–8. 6805625 10.1136/bmj.284.6329.1607 PMC1498516

[17] Pate RR. The evolving definition of physical fitness Quest. 1988;40:174–9.

[18] Chase JD, Conn VS. Meta-analysis of fitness outcomes from motivational physical activity interventions. Nurs Res. 2013;62(5):294–304. doi: 10.1097/NNR.0b013e3182a0395c 23995463 10.1097/NNR.0b013e3182a0395c PMC4629242

[19] Fruin M, Ranking J. Validity of a multi-sensor armband in estimating rest and exercise energy expenditure. Med Sci Sports Exerc. 2004;36(6):1063–9. 15179178 10.1249/01.mss.0000128144.91337.38

[20] Baptista F, Santos DA, Silva AM, Mota J, Santos R, Vale S, et al Prevalence of the portuguese population attaining sufficient physical activity. Med Sci Sports Exerc. 2011;44(3):466–73. 10.1249/MSS.0b013e318230e441 21844823

[21] Ortlieb S, Dias A, Gorzelniak L, Nowak D, Karrasch S, Peters A, et al Exploring patterns of accelerometry-assessed physical activity in elderly people. Int J Behav Nutr Phy. 2014;11(28). 10.1186/1479-5868-11-28 PMC4016218 24575796

[22] Almeida GJM, Wasko MCM, Jeong K, Moore CG, Piva SR. Physical activity measured by the SenseWear armband in women with rheumatoid arthritis. Phys Ther. 2011;91(9):1367–76. doi: 10.2522/ptj.20100291 21719635 10.2522/ptj.20100291 PMC3169787

[23] Godin G. The Godin-Shephard Leisure-Time Physical Activity Questionnaire. ACSM's Health Fit J. 2011;4.

[24] Ball K, Crawford D, Owen N. Too fat to exercise? Obesity as a barrier to physical activity. Aust N Z J Public Health. 2000;24(3):331–3. 10937415 10.1111/j.1467-842x.2000.tb01579.x

[25] Chan CB, Ryan DA. Assessing the effects of weather conditions on physical activity participation using objective measures. Int J Environ Res Public Health. 2009;6(10):2639–54. doi: 10.3390/ijerph6102639 20054460 10.3390/ijerph6102639 PMC2790098

[26] Biddle SJH, Edwardson CL, Wilmot EG, Yates T, Gorely T, Bodicoat DH, et al A randomised controlled trial to reduce sedentary time in young adults at risk of type 2 diabetes mellitus: project STAND (Sedentary Time ANd Diabetes). PLoS ONE. 2015;10(12). 10.1371/journal.pone.0143398 PMC4666612 26623654

[27] Waters L, Reeves M, Fjeldsoe B, Eakin E. Control group improvements in physical activity intervention trials and possible explanatory factors: A systematic review. J Phys Act Health. 2012;9:884–95. 22898467 10.1123/jpah.9.6.884

[28] Gracia-Marco L, Ortega FB, Ruiz JR, Williams CA, Hagströmer M, Manios Y, et al Seasonal variation in physical activity and sedentary time in different European regions. The HELENA study. J Sports Sci. 2013;31(16):1831–40. doi: 10.1080/02640414.2013.803595 24050788 10.1080/02640414.2013.803595

[29] Tomaz SA, Lambert EV, Karpul D, Kolbe-Alexander TL. Cardiovascular fitness is associated with bias between self-reported and objectively measured physical activity. Eur J Sport Sci. 2014;16(1):149–157. doi: 10.1080/17461391.2014.987323 25537282 10.1080/17461391.2014.987323

[30] Lenters-Westra E, Schindhelm RK, Bilo HJ, Groenier KH, Slingerland RJ. Differences in interpretation of haemoglobin A1c values among diabetes care professionals. Neth J Med. 2014;72(9):462–6. 25431391

[31] Bloomgarden ZT, Dodis R, Viscoli CM, Holmboe ES, Inzucchi SE. Lower baseline glycemia reduces apparent oral agent glucose-lowering efficacy. A meta-regression analysis. Diabetes Care. 2006;29(9) 29(9):2137–39. doi: 10.2337/dc06-1120 16936168 10.2337/dc06-1120

[32] Rosner B. Fundamentals of Biostatistics. 4th ed. Duxbury Press; 1995 p. 221.

