# Supplementary material for: Short- and long-term effects of a need-supportive physical activity intervention among patients with type 2 diabetes mellitus: A randomized controlled pilot trial
Source: PLoS One. 2017 Apr 6;12(4):e0174805. doi: 10.1371/journal.pone.0174805 (PMC5383224; doi:10.1371/journal.pone.0174805)
Supplement: S1 Protocol — Note: this protocol was approved by the Medical Ethics Committee of the University Hospitals Leuven before the start of the trial. (PDF) [file pone.0174805.s003.pdf]

Commissie Medische Ethiek/ Klinisch Onderzoek
Faculteit Geneeskunde K.U. Leuven
Centrum voor Klinische Farmacologie
E322 UZ Gasthuisberg
Herestraat 3000 Leuven

*Aanvraag voor advies bij onderzoeksproject*

**ONDERZOEKSPROTOCOL**

**Titel van het onderzoeksproject:**
*'korte en lange termijneffecten van een beweegtraject op de fysieke activiteit, de fysieke fitheid, het subjectief welzijn en de gezondheid bij diabetici'* ***S56117***


**Naam en Functie van de aanvragers**
*Prof. Dr. F. Boen,* Hoofddocent, Faculteit Bewegings- en Revalidatiewetenschappen, Departement Bewegingswetenschappen, Onderzoeksgroep Fysieke Activiteit, Sport en Gezondheid, KU Leuven
*Prof. Dr. J. Seghers*, Hoofddocent, Faculteit Bewegings- en Revalidatiewetenschappen, Departement Bewegingswetenschappen, Onderzoeksgroep Fysieke Activiteit, Sport en Gezondheid, KU Leuven
*Prof. Dr. C. Delecluse*, Gewoon Hoogleraar, Faculteit Bewegings- en Revalidatiewetenschappen, Departement Bewegingswetenschappen, Onderzoeksgroep Fysieke Activiteit, Sport en Gezondheid, KU Leuven
*Dr. A. Bogaerts,* Post-doctoraal medewerker, Faculteit Bewegings- en Revalidatiewetenschappen, KU Leuven
*MS. Karlien Devloo,* Doctoraatsstudent, Faculteit Bewegings- en Revalidatiewetenschappen, Departement Bewegingswetenschappen, Onderzoeksgroep Fysieke Activiteit, Sport en Gezondheid, KU Leuven
*MS. Jari Vanroy*, Doctoraatsstudent, Faculteit Bewegings- en Revalidatiewetenschappen, Departement Bewegingswetenschappen, Onderzoeksgroep Fysieke Activiteit, Sport en Gezondheid, KU Leuven

**Adres van de aanvrager:**
An Bogaerts
Faculteit Bewegings- en Revalidatiewetenschappen
Tervuursevest 101
3001 Leuven


## Rationale:

Regelmatig fysiek actief zijn – op een weloverwegen en doordachte manier- maakt ten volle deel uit van een gezonde levensstijl. Positieve effecten van regelmatige fysieke activiteit hebben zowel betrekking op fysiek vlak als op psychosociaal vlak. Dit geldt zeker ook voor diabetici, die een verhoogde kwetsbaarheid hebben voor bepaalde aandoeningen (hart- en bloedvaten…)

Een hele grote groep mensen vindt de weg echter niet naar het huidige (georganiseerde) bewegingsaanbod. Het aanbod komt mogelijk niet tegemoet aan hun noden en/of de drempel naar dit aanbod is te hoog. Ook bij diabetici merken we deze problematiek. Er zijn heel wat diabetespatiënten die niet voldoende fysiek actief zijn en dus niet kunnen genieten van de voordelen. Bij de doelgroep van diabetici vormt beweging echter een belangrijk onderdeel in de behandeling van de aandoening (Umpierre 2011). Er is dus een dringende nood aan

laagdrempelige beweegprogramma's die diabetici aanzetten tot een voldoende fysiek actieve levensstijl, zowel op korte als op lange termijn.

Bij programma's onder leiding van een bewegingsdeskundige kan deze een individueel aangepast en medisch verantwoord sport- of bewegingsprogramma opstellen en zo de sport- of bewegingsdoelen in goede banen leiden. Een dergelijk programma, dat wordt opgemaakt op basis van need-supportive coaching, omvat haalbare korte en lange termijndoelstellingen. Uit eerdere studies bij fysiek inactieve senioren is al gebleken dat individuele begeleiding door een bewegingsdeskundige zowel op lange als korte termijn tot een actievere levensstijl en een hoger mentaal welbevinden leidt (Opdenacker, et al., 2008; Opdenacker, et al., 2010; Delecluse et al., 2009). Het is belangrijk dat deze beweegprogramma's gesuperviseerd worden door ervaren bewegingsdeskundigen die rekening houden met de voorgeschiedenis van de patiënt, de voorkeuren, de medische toestand ...

Met dit project willen we via een aangepast bewegingsprogramma bij diabetici een positief gedrags- en gezondheidseffect realiseren. De focus ligt hierbij op tertiaire preventie. Voor de recrutering van de deelnemers werken we samen met een ziekenfonds in Vlaanderen nl. de Christelijke Mutualiteit (CM).

## Doelstelling

Met dit project wensen we een methodiek te ontwikkelen om meer beweging te stimuleren bij inactieve diabetici via een aangepast bewegingsprogramma. We ontwikkelen hiervoor een een individueel aangepast en medisch verantwoord beweegplan en trachten de deelnemers te stimuleren om na het stopzetten van de coaching zelfredzaam te zijn om het beweegplan verder te zetten.
Via een gerandomiseerde gecontroleerde wetenschappelijke studie willen we bovendien het gedrags- en gezondheidseffect evalueren op korte en lange termijn. Door middel van deze studie, gecombineerd met een grondige procesevaluatie bij alle stakeholders, willen we verder ook nagaan of het beweegtraject uitrolbaar is (vb. naar andere doelgroepen en/of andere regio's).

## Methoden

### Recrutering

*Doelgroep*

Leden van de doelgroep worden door de CM geselecteerd uit de database van de CM op basis van volgende voorwaarden:

- Diabetes type 2, met minimaal 3 maanden orale diabetica in 2012 met uitsluiting van personen die min. 1 aflevering van Byetta, Victoza of Insuline kregen in 2012
- Aangesloten bij CM Leuven
- Globaal Medisch Dossier
- > 18 jaar

We richten ons met dit project op inactieve diabetici. Dit zijn personen die op het vlak van beweging de gezondheidsnorm (30 minuten matige fysieke activiteit/dag) niet halen en dus niet voldoende fysiek actief zijn.

*Aanschrijven doelgroep*

Leden uit de doelgroep ontvangen een brief waarin het project kort wordt voorgesteld en waarin men wordt uitgenodigd voor een infosessie die gaat over het belang van voldoende beweging voor diabetespatiënten en meer toelichting geeft bij het project. Bij het aanschrijven van de doelgroep wordt er ingespeeld op de positieve sociale identiteit: mensen een unieke

kans geven tot interactie met lotgenotencontact, samen bewegen met andere diabetici met dezelfde beginsituatie, beroep kunnen doen op een individuele bewegingscoach.

*Infosessie*
We opteren om te werken met een kennismakingssessie voor een groot aantal mensen, waaruit dan later verschillende groepen worden gevormd. Tijdens de infosessie komen de voordelen van meer bewegen aan bod, en wordt duidelijk dat het project gaat om bewegen integreren in het dagelijkse leven, en dus niet om sport. Eveneens worden de verschillende facetten van het project voorgesteld, wat er aan deelnemers aangeboden wordt en wat er van deelnemers verwacht wordt. Deelnemers kunnen zich na de infosessie inschrijven voor deelname aan het beweegtraject.

*Screening*
Vooraleer personen kunnen instappen in het begeleidingstraject, dienen zij langs de huisarts te passeren voor goedkeuring. De huisarts zal volgens een vooraf opgesteld screeningsprotocol moeten beslissen of de persoon kan deelnemen aan het project. De huisartsen in de regio zijn voor de start van het project ingelicht over de inhoud van het project en de bijhorende screening, en dit via een brief die vanuit de CM zal vertrekken.

**Begeleiding**
Deelnemers volgen een beweegtraject dat bestaat uit een mix van groepssessies en coaching, met intake en outtake:

*Intake*
Dit gesprek vormt de start van het beweegtraject en duurt 1 uur. We stellen hierbij in overleg met de deelnemer een beweegplan op, aangepast aan de deelnemer. Dit beweegplan bestaat uit activiteiten die in groep onder begeleiding van de bewegingscoach zullen gebeuren en uit activiteiten die in de thuissituatie zullen uitgevoerd worden.
De need-supportive coaching speelt in op de psychologische basisbehoeften: autonomie, relationele verbondenheid en de competentie van de deelnemer.

*Groepslessen*
Gedurende vijf weken neemt de deelnemer deel aan groepslessen. Deze bieden extra ondersteuning voor deelnemers en zullen de therapietrouw bevorderen. Het oefenprogramma wordt in kleine groepjes begeleid (gemiddeld 5 personen) door een bewegingscoach (Master in de Bewegingswetenschappen met afstudeerrichting Fysieke Activiteit, Fitness en Gezondheid) en bestaat uit laagdrempelige bewegingsactiviteiten (vb. wandelen, Nordic Walking …). De groepslessen vinden (zoveel mogelijk) plaats in de nabijheid van de deelnemer.

*Outtake*
Na het beweegtraject wordt opnieuw een individueel contactmoment tussen bewegingscoach en deelnemer voorzien. Hierbij wordt het beweegplan verder op punt gesteld, met het oog op een zelfredzame en actieve levensstijl.

## **Onderzoek**
### Onderzoeksvragen
- M.b.t. gedrags- en gezondheidseffect:

  - In welke mate heeft de interventie op korte en lange termijn effecten m.b.t. gedrag?
  - In welke mate heeft de interventie op korte en lange termijn effecten m.b.t. gezondheid?
- M.b.t. proces:
  - Hoe ervaren deelnemers de verschillende elementen van het traject (brief, infosessie, coaching, groepssessies …)
  - In welke mate zijn leden uit de doelgroep geïnteresseerd in deze dienstverlening aan een reële prijs?
  - Welke lessen zijn te trekken uit de gebruikte communicatie naar doelgroep en huisartsen?

**Onderzoeksopzet**

In het onderzoek wordt gewerkt met een interventie- en een controlegroep. De interventiegroep zal bestaan uit 60 CM-leden die voldoen aan de voorwaarden van de doelgroep. De controlegroep zal bestaan uit 40 CM-leden die voldoen aan de voorwaarden. Zij opereren als waiting groep, wat betekent dat zij tijdens de eerste zes maanden van het project enkel als controlegroep dienen en nog geen interventie ondergaan (voor een overzicht, zie 'timing'). Zowel de interventiegroep als de controlegroep zullen op vaste tijdstippen metingen ondergaan:

- De interventiegroep legt metingen af in de pretest (start), posttest (na 6 weken), follow up 1 (na 6 maanden) en follow up 2 (na 12 maanden).
- De controlegroep legt metingen af in de baseline 1 test (start), baseline 2 test (na 6 weken), pretest (na 6 maanden), posttest (na 6 maanden + 6 weken).

We streven ernaar om de metingen bij de huisarts te laten vallen binnen de reguliere driemaandelijkse consulten voor diabetespatiënten.

***Meting gedrag en fysieke fitheid***

Het meten van mogelijke effecten in gedrag zal gebeuren aan de hand van volgende elementen:

- Psychologische vragenlijsten
- Subjectieve vragenlijsten omtrent fysieke activiteit en zitgedrag
- Objectieve monitoring van het beweeggedrag via beweegmonitor (SenseWear) die gedurende enkele dagen aan de bovenarm wordt gedragen
- De fysieke fitheid wordt geëvalueerd aan de hand van een laagdrempelige submaximale inspanningstest nl de 6-minuten wandeltest. Dit is een wetenschappelijke gevalideerde test waarbij de deelnemers gedurende 6 minuten zoveel mogelijk afstand moeten afleggen al wandelend en waarbij de hartslag wordt opgevolgd.

***Meting gezondheid***

De effecten in gezondheid worden gemeten door de huisarts. Deelnemers ontvangen hiervoor een boekje waarin de huisarts deze gegevens kan opschrijven. We verzamelen vervolgens deze resultaten. De huisarts registreert volgende elementen:

- Gewicht
- Bloeddruk
- HbA1C bloedwaarde

Zowel bij het meten van gezondheid als bij het meten van gedrag zal er dus gewerkt worden met een interventie- en controlegroep.

***Procesevaluatie***

Hiertoe zal bij deelnemers bevraagd worden hoe zij het gehele proces met zijn verschillende onderdelen ervaren hebben. Ook bij huisartsen en andere stakeholders kan bevraagd worden hoe zij proces en de communicatie ervaren hebben en wat zij van het initiatief vinden.

## Timing

| Fase | Inhoud | Periode |
|---|---|---|
| **Fase 0** | **Voorbereidingen project** | Oktober – November 2013 |
| **Fase 1** | **Kick-off stuurgroep** | November 2013 |
| **Fase 2** | **Communicatie naar huisartsen** | December 2013 |
| **Fase 3** | **Communicatie naar doelgroep**<br>- Communicatie naar interventiegroep<br>- Communicatie naar controlegroep | Januari – februari 2014 |
| **Fase 4** | **Infosessies en inschrijvingen** | Februari – maart 2014 |
| **Fase 5** | **Screening, pretest, beweegtraject en posttest**<br>Interventiegroep:<br>- Screening  en pretest (huisarts + FaBeR)<br>- 6 weken beweegtraject<br>- Posttest (huisarts + FaBeR)<br>Controlegroep:<br>- Baseline1-test controlegroep<br>- Baseline2-test controlegroep 6 weken later | Maart – juli 2014 |
| **Fase 6** | **Follow-up en beweegtraject voor controlegroep**<br>Interventiegroep:<br>- Follow-up 1 bij huisarts en Portaal<br>Controlegroep:<br>- Screening en pretest (FaBeR en huisarts)<br>- 6 weken beweegtraject<br>- Posttest (FaBeR en huisarts) | September 2014 – januari 2015 |
| **Fase 7** | **Tussentijdse rapportering** | Februari – maart 2015 |
| **Fase 8** | **Follow-up 2**<br>Interventiegroep:<br>- Follow-up 2 bij huisarts en Portaal | |
| **Fase 9** | **Eindrapportering** | Juni – augustus 2015 |
