# Supplementary material for: Short- and long-term effects of a need-supportive physical activity intervention among patients with type 2 diabetes mellitus: A randomized controlled pilot trial
Source: PLoS One. 2017 Apr 6;12(4):e0174805. doi: 10.1371/journal.pone.0174805 (PMC5383224; doi:10.1371/journal.pone.0174805)
Supplement: S1 Supporting information — SPSS full models and output. (DOC) [file pone.0174805.s005.doc]

MIXED HbA1C_Lgx1 BY Groep Tijd WITH Leeftijd Geslacht VLt2EU VLt2SU
  /CRITERIA=CIN(95) MXITER(100) MXSTEP(10) SCORING(1) SINGULAR(0.000000000001) HCONVERGE(0,
    ABSOLUTE) LCONVERGE(0, ABSOLUTE) PCONVERGE(0.000001, ABSOLUTE)
  /FIXED=Tijd Groep*Tijd Groep Leeftijd Geslacht VLt2EU VLt2SU | SSTYPE(3)
  /METHOD=REML
  /REPEATED=Tijd | SUBJECT(Volgnummer) COVTYPE(UN).


Mixed Model Analysis


Notes	
Output Created	09-FEB-2017 10:23:28	
Comments		
Input	Data	C:\Users\u0093379\Documents\Onderzoek\PhD\CM\Deelnemers BIBt1t2_long format.sav	
	Active Dataset	DataSet1	
	Filter	<none>	
	Weight	<none>	
	Split File	<none>	
	N of Rows in Working Data File	96	
Missing Value Handling	Definition of Missing	User-defined missing values are treated as missing.	
	Cases Used	Statistics are based on all cases with valid data for all variables in the model.	
Syntax	MIXED HbA1C_Lgx1 BY Groep Tijd WITH Leeftijd Geslacht VLt2EU VLt2SU
  /CRITERIA=CIN(95) MXITER(100) MXSTEP(10) SCORING(1) SINGULAR(0.000000000001) HCONVERGE(0,
    ABSOLUTE) LCONVERGE(0, ABSOLUTE) PCONVERGE(0.000001, ABSOLUTE)
  /FIXED=Tijd Groep*Tijd Groep Leeftijd Geslacht VLt2EU VLt2SU | SSTYPE(3)
  /METHOD=REML
  /REPEATED=Tijd | SUBJECT(Volgnummer) COVTYPE(UN).	
Resources	Processor Time	00:00:00,02	
	Elapsed Time	00:00:00,05	


[DataSet1] C:\Users\u0093379\Documents\Onderzoek\PhD\CM\Deelnemers BIBt1t2_long format.sav


Model Dimensiona	
	Number of Levels	Covariance Structure	Number of Parameters	Subject Variables	
Fixed Effects	Intercept	1		1		
	Tijd	2		1		
	Groep * Tijd	4		2		
	Groep	2		0		
	Leeftijd	1		1		
	Geslacht	1		1		
	VLt2EU	1		1		
	VLt2SU	1		1		
Repeated Effects	Tijd	2	Unstructured	3	Volgnummer	
Total	15		11		

Model Dimensiona	
	Number of Subjects	
Fixed Effects	Intercept		
	Tijd		
	Groep * Tijd		
	Groep		
	Leeftijd		
	Geslacht		
	VLt2EU		
	VLt2SU		
Repeated Effects	Tijd	39	
Total		

a. Dependent Variable: HbA1C_Lgx1.	


Information Criteriaa	
-2 Restricted Log Likelihood	-275,671	
Akaike's Information Criterion (AIC)	-269,671	
Hurvich and Tsai's Criterion (AICC)	-269,302	
Bozdogan's Criterion (CAIC)	-259,969	
Schwarz's Bayesian Criterion (BIC)	-262,969	

The information criteria are displayed in smaller-is-better form.a	
a. Dependent Variable: HbA1C_Lgx1.	


Fixed Effects


Type III Tests of Fixed Effectsa	
Source	Numerator df	Denominator df	F	Sig.	
Intercept	1	33,015	346,639	,000	
Tijd	1	35,988	8,543	,006	
Groep * Tijd	1	35,988	,991	,326	
Groep	1	32,660	,186	,669	
Leeftijd	1	32,970	,297	,589	
Geslacht	1	32,978	,537	,469	
VLt2EU	1	32,984	2,219	,146	
VLt2SU	1	32,970	,350	,558	

a. Dependent Variable: HbA1C_Lgx1.	


Covariance Parameters


Estimates of Covariance Parametersa	
Parameter	Estimate	Std. Error	
Repeated Measures	UN (1,1)	,001712	,000449	
	UN (2,1)	,001336	,000362	
	UN (2,2)	,001384	,000341	

a. Dependent Variable: HbA1C_Lgx1.	


MIXED HbA1C_Lgx1 BY Groep Tijd
  /CRITERIA=CIN(95) MXITER(100) MXSTEP(10) SCORING(1) SINGULAR(0.000000000001) HCONVERGE(0,
    ABSOLUTE) LCONVERGE(0, ABSOLUTE) PCONVERGE(0.000001, ABSOLUTE)
  /FIXED=Tijd Groep*Tijd Groep | SSTYPE(3)
  /METHOD=REML
  /REPEATED=Tijd | SUBJECT(Volgnummer) COVTYPE(UN).


Mixed Model Analysis


Notes	
Output Created	09-FEB-2017 10:23:28	
Comments		
Input	Data	C:\Users\u0093379\Documents\Onderzoek\PhD\CM\Deelnemers BIBt1t2_long format.sav	
	Active Dataset	DataSet1	
	Filter	<none>	
	Weight	<none>	
	Split File	<none>	
	N of Rows in Working Data File	96	
Missing Value Handling	Definition of Missing	User-defined missing values are treated as missing.	
	Cases Used	Statistics are based on all cases with valid data for all variables in the model.	
Syntax	MIXED HbA1C_Lgx1 BY Groep Tijd
  /CRITERIA=CIN(95) MXITER(100) MXSTEP(10) SCORING(1) SINGULAR(0.000000000001) HCONVERGE(0,
    ABSOLUTE) LCONVERGE(0, ABSOLUTE) PCONVERGE(0.000001, ABSOLUTE)
  /FIXED=Tijd Groep*Tijd Groep | SSTYPE(3)
  /METHOD=REML
  /REPEATED=Tijd | SUBJECT(Volgnummer) COVTYPE(UN).	
Resources	Processor Time	00:00:00,03	
	Elapsed Time	00:00:00,04	


Model Dimensiona	
	Number of Levels	Covariance Structure	Number of Parameters	Subject Variables	
Fixed Effects	Intercept	1		1		
	Tijd	2		1		
	Groep * Tijd	4		2		
	Groep	2		0		
Repeated Effects	Tijd	2	Unstructured	3	Volgnummer	
Total	11		7		

Model Dimensiona	
	Number of Subjects	
Fixed Effects	Intercept		
	Tijd		
	Groep * Tijd		
	Groep		
Repeated Effects	Tijd	48	
Total		

a. Dependent Variable: HbA1C_Lgx1.	


Information Criteriaa	
-2 Restricted Log Likelihood	-336,861	
Akaike's Information Criterion (AIC)	-330,861	
Hurvich and Tsai's Criterion (AICC)	-330,561	
Bozdogan's Criterion (CAIC)	-320,568	
Schwarz's Bayesian Criterion (BIC)	-323,568	

The information criteria are displayed in smaller-is-better form.a	
a. Dependent Variable: HbA1C_Lgx1.	


Fixed Effects


Type III Tests of Fixed Effectsa	
Source	Numerator df	Denominator df	F	Sig.	
Intercept	1	44,529	21126,932	,000	
Tijd	1	37,278	10,128	,003	
Groep * Tijd	1	37,278	,627	,434	
Groep	1	44,529	,129	,721	

a. Dependent Variable: HbA1C_Lgx1.	


Covariance Parameters


Estimates of Covariance Parametersa	
Parameter	Estimate	Std. Error	
Repeated Measures	UN (1,1)	,002018	,000423	
	UN (2,1)	,001610	,000369	
	UN (2,2)	,001639	,000371	

a. Dependent Variable: HbA1C_Lgx1.	

MIXED ZMWTafstand BY Groep Tijd WITH Geslacht Leeftijd BMIbaseline VerschilBMIt2t1
  /CRITERIA=CIN(95) MXITER(100) MXSTEP(10) SCORING(1) SINGULAR(0.000000000001) HCONVERGE(0,
    ABSOLUTE) LCONVERGE(0, ABSOLUTE) PCONVERGE(0.000001, ABSOLUTE)
  /FIXED=Groep Tijd Geslacht Leeftijd BMIbaseline VerschilBMIt2t1 Groep*Tijd | SSTYPE(3)
  /METHOD=REML
  /REPEATED=Tijd | SUBJECT(Volgnummer) COVTYPE(UN).


Mixed Model Analysis


Notes	
Output Created	09-FEB-2017 10:24:17	
Comments		
Input	Data	C:\Users\u0093379\Documents\Onderzoek\PhD\CM\Deelnemers BIBt1t2_long format.sav	
	Active Dataset	DataSet1	
	Filter	<none>	
	Weight	<none>	
	Split File	<none>	
	N of Rows in Working Data File	96	
Missing Value Handling	Definition of Missing	User-defined missing values are treated as missing.	
	Cases Used	Statistics are based on all cases with valid data for all variables in the model.	
Syntax	MIXED ZMWTafstand BY Groep Tijd WITH Geslacht Leeftijd BMIbaseline VerschilBMIt2t1
  /CRITERIA=CIN(95) MXITER(100) MXSTEP(10) SCORING(1) SINGULAR(0.000000000001) HCONVERGE(0,
    ABSOLUTE) LCONVERGE(0, ABSOLUTE) PCONVERGE(0.000001, ABSOLUTE)
  /FIXED=Groep Tijd Geslacht Leeftijd BMIbaseline VerschilBMIt2t1 Groep*Tijd | SSTYPE(3)
  /METHOD=REML
  /REPEATED=Tijd | SUBJECT(Volgnummer) COVTYPE(UN).	
Resources	Processor Time	00:00:00,03	
	Elapsed Time	00:00:00,09	


Model Dimensiona	
	Number of Levels	Covariance Structure	Number of Parameters	
Fixed Effects	Intercept	1		1	
	Groep	2		1	
	Tijd	2		1	
	Geslacht	1		1	
	Leeftijd	1		1	
	BMIbaseline	1		1	
	VerschilBMIt2t1	1		1	
	Groep * Tijd	4		1	
Repeated Effects	Tijd	2	Unstructured	3	
Total	15		11	

Model Dimensiona	
	Subject Variables	Number of Subjects	
Fixed Effects	Intercept			
	Groep			
	Tijd			
	Geslacht			
	Leeftijd			
	BMIbaseline			
	VerschilBMIt2t1			
	Groep * Tijd			
Repeated Effects	Tijd	Volgnummer	42	
Total			

a. Dependent Variable: ZMWTafstand.	


Information Criteriaa	
-2 Restricted Log Likelihood	790,099	
Akaike's Information Criterion (AIC)	796,099	
Hurvich and Tsai's Criterion (AICC)	796,447	
Bozdogan's Criterion (CAIC)	805,971	
Schwarz's Bayesian Criterion (BIC)	802,971	

The information criteria are displayed in smaller-is-better form.a	
a. Dependent Variable: ZMWTafstand.	


Fixed Effects


Type III Tests of Fixed Effectsa	
Source	Numerator df	Denominator df	F	Sig.	
Intercept	1	35,882	77,948	,000	
Groep	1	35,963	,004	,949	
Tijd	1	37,072	2,913	,096	
Geslacht	1	35,989	3,998	,053	
Leeftijd	1	35,864	19,718	,000	
BMIbaseline	1	35,875	2,176	,149	
VerschilBMIt2t1	1	35,913	,106	,746	
Groep * Tijd	1	37,075	,061	,806	

a. Dependent Variable: ZMWTafstand.	


Covariance Parameters


Estimates of Covariance Parametersa	
Parameter	Estimate	Std. Error	
Repeated Measures	UN (1,1)	4885,196090	1151,705161	
	UN (2,1)	4611,756183	1138,040130	
	UN (2,2)	5112,078499	1222,847112	

a. Dependent Variable: ZMWTafstand.	


MIXED ZMWTafstand BY Groep Tijd
  /CRITERIA=CIN(95) MXITER(100) MXSTEP(10) SCORING(1) SINGULAR(0.000000000001) HCONVERGE(0,
    ABSOLUTE) LCONVERGE(0, ABSOLUTE) PCONVERGE(0.000001, ABSOLUTE)
  /FIXED=Groep Tijd Groep*Tijd | SSTYPE(3)
  /METHOD=REML
  /REPEATED=Tijd | SUBJECT(Volgnummer) COVTYPE(UN).


Mixed Model Analysis


Notes	
Output Created	09-FEB-2017 10:24:18	
Comments		
Input	Data	C:\Users\u0093379\Documents\Onderzoek\PhD\CM\Deelnemers BIBt1t2_long format.sav	
	Active Dataset	DataSet1	
	Filter	<none>	
	Weight	<none>	
	Split File	<none>	
	N of Rows in Working Data File	96	
Missing Value Handling	Definition of Missing	User-defined missing values are treated as missing.	
	Cases Used	Statistics are based on all cases with valid data for all variables in the model.	
Syntax	MIXED ZMWTafstand BY Groep Tijd
  /CRITERIA=CIN(95) MXITER(100) MXSTEP(10) SCORING(1) SINGULAR(0.000000000001) HCONVERGE(0,
    ABSOLUTE) LCONVERGE(0, ABSOLUTE) PCONVERGE(0.000001, ABSOLUTE)
  /FIXED=Groep Tijd Groep*Tijd | SSTYPE(3)
  /METHOD=REML
  /REPEATED=Tijd | SUBJECT(Volgnummer) COVTYPE(UN).	
Resources	Processor Time	00:00:00,02	
	Elapsed Time	00:00:00,06	


Model Dimensiona	
	Number of Levels	Covariance Structure	Number of Parameters	Subject Variables	
Fixed Effects	Intercept	1		1		
	Groep	2		1		
	Tijd	2		1		
	Groep * Tijd	4		1		
Repeated Effects	Tijd	2	Unstructured	3	Volgnummer	
Total	11		7		

Model Dimensiona	
	Number of Subjects	
Fixed Effects	Intercept		
	Groep		
	Tijd		
	Groep * Tijd		
Repeated Effects	Tijd	43	
Total		

a. Dependent Variable: ZMWTafstand.	


Information Criteriaa	
-2 Restricted Log Likelihood	842,873	
Akaike's Information Criterion (AIC)	848,873	
Hurvich and Tsai's Criterion (AICC)	849,197	
Bozdogan's Criterion (CAIC)	858,943	
Schwarz's Bayesian Criterion (BIC)	855,943	

The information criteria are displayed in smaller-is-better form.a	
a. Dependent Variable: ZMWTafstand.	


Fixed Effects


Type III Tests of Fixed Effectsa	
Source	Numerator df	Denominator df	F	Sig.	
Intercept	1	41,117	1633,588	,000	
Groep	1	41,117	,256	,616	
Tijd	1	37,067	3,227	,081	
Groep * Tijd	1	37,067	,076	,784	

a. Dependent Variable: ZMWTafstand.	


Covariance Parameters


Estimates of Covariance Parametersa	
Parameter	Estimate	Std. Error	
Repeated Measures	UN (1,1)	7110,909926	1570,537270	
	UN (2,1)	7304,677034	1655,514686	
	UN (2,2)	8271,641281	1833,169331	

a. Dependent Variable: ZMWTafstand.	


MIXED PAtotaal_Lgx1 BY Groep Tijd WITH Geslacht Leeftijd BMIbaseline VerschilBMIt2t1 Daglengtet2 Temperatuurt2 Neerslagt2
  /CRITERIA=CIN(95) MXITER(100) MXSTEP(10) SCORING(1) SINGULAR(0.000000000001) HCONVERGE(0,
    ABSOLUTE) LCONVERGE(0, ABSOLUTE) PCONVERGE(0.000001, ABSOLUTE)
  /FIXED=Groep Tijd Geslacht Leeftijd BMIbaseline VerschilBMIt2t1 Daglengtet2 Temperatuurt2 Neerslagt2 Groep*Tijd | SSTYPE(3)
  /METHOD=REML
  /REPEATED=Tijd | SUBJECT(Volgnummer) COVTYPE(UN).


Mixed Model Analysis


Notes	
Output Created	09-FEB-2017 10:24:18	
Comments		
Input	Data	C:\Users\u0093379\Documents\Onderzoek\PhD\CM\Deelnemers BIBt1t2_long format.sav	
	Active Dataset	DataSet1	
	Filter	<none>	
	Weight	<none>	
	Split File	<none>	
	N of Rows in Working Data File	96	
Missing Value Handling	Definition of Missing	User-defined missing values are treated as missing.	
	Cases Used	Statistics are based on all cases with valid data for all variables in the model.	
Syntax	MIXED PAtotaal_Lgx1 BY Groep Tijd WITH Geslacht Leeftijd BMIbaseline VerschilBMIt2t1 Daglengtet2 Temperatuurt2 Neerslagt2
  /CRITERIA=CIN(95) MXITER(100) MXSTEP(10) SCORING(1) SINGULAR(0.000000000001) HCONVERGE(0,
    ABSOLUTE) LCONVERGE(0, ABSOLUTE) PCONVERGE(0.000001, ABSOLUTE)
  /FIXED=Groep Tijd Geslacht Leeftijd BMIbaseline VerschilBMIt2t1 Daglengtet2 Temperatuurt2 Neerslagt2 Groep*Tijd | SSTYPE(3)
  /METHOD=REML
  /REPEATED=Tijd | SUBJECT(Volgnummer) COVTYPE(UN).	
Resources	Processor Time	00:00:00,02	
	Elapsed Time	00:00:00,05	


Model Dimensiona	
	Number of Levels	Covariance Structure	Number of Parameters	
Fixed Effects	Intercept	1		1	
	Groep	2		1	
	Tijd	2		1	
	Geslacht	1		1	
	Leeftijd	1		1	
	BMIbaseline	1		1	
	VerschilBMIt2t1	1		1	
	Daglengtet2	1		1	
	Temperatuurt2	1		1	
	Neerslagt2	1		1	
	Groep * Tijd	4		1	
Repeated Effects	Tijd	2	Unstructured	3	
Total	18		14	

Model Dimensiona	
	Subject Variables	Number of Subjects	
Fixed Effects	Intercept			
	Groep			
	Tijd			
	Geslacht			
	Leeftijd			
	BMIbaseline			
	VerschilBMIt2t1			
	Daglengtet2			
	Temperatuurt2			
	Neerslagt2			
	Groep * Tijd			
Repeated Effects	Tijd	Volgnummer	44	
Total			

a. Dependent Variable: PAtotaal_Lgx1.	


Information Criteriaa	
-2 Restricted Log Likelihood	56,408	
Akaike's Information Criterion (AIC)	62,408	
Hurvich and Tsai's Criterion (AICC)	62,766	
Bozdogan's Criterion (CAIC)	72,196	
Schwarz's Bayesian Criterion (BIC)	69,196	

The information criteria are displayed in smaller-is-better form.a	
a. Dependent Variable: PAtotaal_Lgx1.	


Fixed Effects


Type III Tests of Fixed Effectsa	
Source	Numerator df	Denominator df	F	Sig.	
Intercept	1	33,435	4,287	,046	
Groep	1	34,924	,167	,686	
Tijd	1	36,905	4,503	,041	
Geslacht	1	35,017	,354	,556	
Leeftijd	1	34,485	,381	,541	
BMIbaseline	1	34,060	,082	,776	
VerschilBMIt2t1	1	37,386	,802	,376	
Daglengtet2	1	34,007	,066	,799	
Temperatuurt2	1	34,730	,263	,612	
Neerslagt2	1	33,368	,088	,769	
Groep * Tijd	1	36,821	,531	,471	

a. Dependent Variable: PAtotaal_Lgx1.	


Covariance Parameters


Estimates of Covariance Parametersa	
Parameter	Estimate	Std. Error	
Repeated Measures	UN (1,1)	,090517	,021553	
	UN (2,1)	,060058	,019015	
	UN (2,2)	,088982	,022653	

a. Dependent Variable: PAtotaal_Lgx1.	


MIXED PAtotaal_Lgx1 BY Groep Tijd
  /CRITERIA=CIN(95) MXITER(100) MXSTEP(10) SCORING(1) SINGULAR(0.000000000001) HCONVERGE(0,
    ABSOLUTE) LCONVERGE(0, ABSOLUTE) PCONVERGE(0.000001, ABSOLUTE)
  /FIXED=Groep Tijd Groep*Tijd | SSTYPE(3)
  /METHOD=REML
  /REPEATED=Tijd | SUBJECT(Volgnummer) COVTYPE(UN).


Mixed Model Analysis


Notes	
Output Created	09-FEB-2017 10:24:18	
Comments		
Input	Data	C:\Users\u0093379\Documents\Onderzoek\PhD\CM\Deelnemers BIBt1t2_long format.sav	
	Active Dataset	DataSet1	
	Filter	<none>	
	Weight	<none>	
	Split File	<none>	
	N of Rows in Working Data File	96	
Missing Value Handling	Definition of Missing	User-defined missing values are treated as missing.	
	Cases Used	Statistics are based on all cases with valid data for all variables in the model.	
Syntax	MIXED PAtotaal_Lgx1 BY Groep Tijd
  /CRITERIA=CIN(95) MXITER(100) MXSTEP(10) SCORING(1) SINGULAR(0.000000000001) HCONVERGE(0,
    ABSOLUTE) LCONVERGE(0, ABSOLUTE) PCONVERGE(0.000001, ABSOLUTE)
  /FIXED=Groep Tijd Groep*Tijd | SSTYPE(3)
  /METHOD=REML
  /REPEATED=Tijd | SUBJECT(Volgnummer) COVTYPE(UN).	
Resources	Processor Time	00:00:00,02	
	Elapsed Time	00:00:00,04	


Model Dimensiona	
	Number of Levels	Covariance Structure	Number of Parameters	Subject Variables	
Fixed Effects	Intercept	1		1		
	Groep	2		1		
	Tijd	2		1		
	Groep * Tijd	4		1		
Repeated Effects	Tijd	2	Unstructured	3	Volgnummer	
Total	11		7		

Model Dimensiona	
	Number of Subjects	
Fixed Effects	Intercept		
	Groep		
	Tijd		
	Groep * Tijd		
Repeated Effects	Tijd	46	
Total		

a. Dependent Variable: PAtotaal_Lgx1.	


Information Criteriaa	
-2 Restricted Log Likelihood	26,150	
Akaike's Information Criterion (AIC)	32,150	
Hurvich and Tsai's Criterion (AICC)	32,466	
Bozdogan's Criterion (CAIC)	42,296	
Schwarz's Bayesian Criterion (BIC)	39,296	

The information criteria are displayed in smaller-is-better form.a	
a. Dependent Variable: PAtotaal_Lgx1.	


Fixed Effects


Type III Tests of Fixed Effectsa	
Source	Numerator df	Denominator df	F	Sig.	
Intercept	1	40,060	1245,171	,000	
Groep	1	40,060	,037	,848	
Tijd	1	35,199	6,172	,018	
Groep * Tijd	1	35,199	,141	,709	

a. Dependent Variable: PAtotaal_Lgx1.	


Covariance Parameters


Estimates of Covariance Parametersa	
Parameter	Estimate	Std. Error	
Repeated Measures	UN (1,1)	,098817	,021164	
	UN (2,1)	,057467	,017758	
	UN (2,2)	,079281	,019701	

a. Dependent Variable: PAtotaal_Lgx1.	


MIXED SWTPA BY Groep Tijd WITH Geslacht Leeftijd BMIbaseline VerschilBMIt2t1 Daglengtet2 Temperatuurt2 Neerslagt2
  /CRITERIA=CIN(95) MXITER(100) MXSTEP(10) SCORING(1) SINGULAR(0.000000000001) HCONVERGE(0,
    ABSOLUTE) LCONVERGE(0, ABSOLUTE) PCONVERGE(0.000001, ABSOLUTE)
  /FIXED=Groep Tijd Geslacht Leeftijd BMIbaseline VerschilBMIt2t1 Daglengtet2 Temperatuurt2 Neerslagt2 Groep*Tijd | SSTYPE(3)
  /METHOD=REML
  /REPEATED=Tijd | SUBJECT(Volgnummer) COVTYPE(UN).


Mixed Model Analysis


Notes	
Output Created	09-FEB-2017 10:24:18	
Comments		
Input	Data	C:\Users\u0093379\Documents\Onderzoek\PhD\CM\Deelnemers BIBt1t2_long format.sav	
	Active Dataset	DataSet1	
	Filter	<none>	
	Weight	<none>	
	Split File	<none>	
	N of Rows in Working Data File	96	
Missing Value Handling	Definition of Missing	User-defined missing values are treated as missing.	
	Cases Used	Statistics are based on all cases with valid data for all variables in the model.	
Syntax	MIXED SWTPA BY Groep Tijd WITH Geslacht Leeftijd BMIbaseline VerschilBMIt2t1 Daglengtet2 Temperatuurt2 Neerslagt2
  /CRITERIA=CIN(95) MXITER(100) MXSTEP(10) SCORING(1) SINGULAR(0.000000000001) HCONVERGE(0,
    ABSOLUTE) LCONVERGE(0, ABSOLUTE) PCONVERGE(0.000001, ABSOLUTE)
  /FIXED=Groep Tijd Geslacht Leeftijd BMIbaseline VerschilBMIt2t1 Daglengtet2 Temperatuurt2 Neerslagt2 Groep*Tijd | SSTYPE(3)
  /METHOD=REML
  /REPEATED=Tijd | SUBJECT(Volgnummer) COVTYPE(UN).	
Resources	Processor Time	00:00:00,05	
	Elapsed Time	00:00:00,07	


Model Dimensiona	
	Number of Levels	Covariance Structure	Number of Parameters	
Fixed Effects	Intercept	1		1	
	Groep	2		1	
	Tijd	2		1	
	Geslacht	1		1	
	Leeftijd	1		1	
	BMIbaseline	1		1	
	VerschilBMIt2t1	1		1	
	Daglengtet2	1		1	
	Temperatuurt2	1		1	
	Neerslagt2	1		1	
	Groep * Tijd	4		1	
Repeated Effects	Tijd	2	Unstructured	3	
Total	18		14	

Model Dimensiona	
	Subject Variables	Number of Subjects	
Fixed Effects	Intercept			
	Groep			
	Tijd			
	Geslacht			
	Leeftijd			
	BMIbaseline			
	VerschilBMIt2t1			
	Daglengtet2			
	Temperatuurt2			
	Neerslagt2			
	Groep * Tijd			
Repeated Effects	Tijd	Volgnummer	44	
Total			

a. Dependent Variable: SWTPA.	


Information Criteriaa	
-2 Restricted Log Likelihood	815,686	
Akaike's Information Criterion (AIC)	821,686	
Hurvich and Tsai's Criterion (AICC)	822,055	
Bozdogan's Criterion (CAIC)	831,388	
Schwarz's Bayesian Criterion (BIC)	828,388	

The information criteria are displayed in smaller-is-better form.a	
a. Dependent Variable: SWTPA.	


Fixed Effects


Type III Tests of Fixed Effectsa	
Source	Numerator df	Denominator df	F	Sig.	
Intercept	1	34,117	11,590	,002	
Groep	1	33,104	,088	,768	
Tijd	1	35,696	,111	,741	
Geslacht	1	34,433	7,414	,010	
Leeftijd	1	32,337	9,422	,004	
BMIbaseline	1	34,196	1,066	,309	
VerschilBMIt2t1	1	32,132	,486	,491	
Daglengtet2	1	33,989	,001	,975	
Temperatuurt2	1	35,255	,050	,825	
Neerslagt2	1	35,899	,956	,335	
Groep * Tijd	1	35,983	,435	,514	

a. Dependent Variable: SWTPA.	


Covariance Parameters


Estimates of Covariance Parametersa	
Parameter	Estimate	Std. Error	
Repeated Measures	UN (1,1)	4711,712851	1121,400292	
	UN (2,1)	2668,920413	993,624833	
	UN (2,2)	4875,926665	1270,065283	

a. Dependent Variable: SWTPA.	


MIXED SWTPA BY Groep Tijd
  /CRITERIA=CIN(95) MXITER(100) MXSTEP(10) SCORING(1) SINGULAR(0.000000000001) HCONVERGE(0,
    ABSOLUTE) LCONVERGE(0, ABSOLUTE) PCONVERGE(0.000001, ABSOLUTE)
  /FIXED=Groep Tijd Groep*Tijd | SSTYPE(3)
  /METHOD=REML
  /REPEATED=Tijd | SUBJECT(Volgnummer) COVTYPE(UN).


Mixed Model Analysis


Notes	
Output Created	09-FEB-2017 10:24:18	
Comments		
Input	Data	C:\Users\u0093379\Documents\Onderzoek\PhD\CM\Deelnemers BIBt1t2_long format.sav	
	Active Dataset	DataSet1	
	Filter	<none>	
	Weight	<none>	
	Split File	<none>	
	N of Rows in Working Data File	96	
Missing Value Handling	Definition of Missing	User-defined missing values are treated as missing.	
	Cases Used	Statistics are based on all cases with valid data for all variables in the model.	
Syntax	MIXED SWTPA BY Groep Tijd
  /CRITERIA=CIN(95) MXITER(100) MXSTEP(10) SCORING(1) SINGULAR(0.000000000001) HCONVERGE(0,
    ABSOLUTE) LCONVERGE(0, ABSOLUTE) PCONVERGE(0.000001, ABSOLUTE)
  /FIXED=Groep Tijd Groep*Tijd | SSTYPE(3)
  /METHOD=REML
  /REPEATED=Tijd | SUBJECT(Volgnummer) COVTYPE(UN).	
Resources	Processor Time	00:00:00,02	
	Elapsed Time	00:00:00,07	


Model Dimensiona	
	Number of Levels	Covariance Structure	Number of Parameters	Subject Variables	
Fixed Effects	Intercept	1		1		
	Groep	2		1		
	Tijd	2		1		
	Groep * Tijd	4		1		
Repeated Effects	Tijd	2	Unstructured	3	Volgnummer	
Total	11		7		

Model Dimensiona	
	Number of Subjects	
Fixed Effects	Intercept		
	Groep		
	Tijd		
	Groep * Tijd		
Repeated Effects	Tijd	46	
Total		

a. Dependent Variable: SWTPA.	


Information Criteriaa	
-2 Restricted Log Likelihood	895,966	
Akaike's Information Criterion (AIC)	901,966	
Hurvich and Tsai's Criterion (AICC)	902,290	
Bozdogan's Criterion (CAIC)	912,036	
Schwarz's Bayesian Criterion (BIC)	909,036	

The information criteria are displayed in smaller-is-better form.a	
a. Dependent Variable: SWTPA.	


Fixed Effects


Type III Tests of Fixed Effectsa	
Source	Numerator df	Denominator df	F	Sig.	
Intercept	1	41,923	117,807	,000	
Groep	1	41,923	,208	,651	
Tijd	1	35,851	,117	,734	
Groep * Tijd	1	35,851	,195	,661	

a. Dependent Variable: SWTPA.	


Covariance Parameters


Estimates of Covariance Parametersa	
Parameter	Estimate	Std. Error	
Repeated Measures	UN (1,1)	6077,552373	1314,266191	
	UN (2,1)	4180,806961	1226,731114	
	UN (2,2)	6545,197436	1566,529029	

a. Dependent Variable: SWTPA.	
