# Supplementary material for: Short- and long-term effects of a need-supportive physical activity intervention among patients with type 2 diabetes mellitus: A randomized controlled pilot trial
Source: PLoS One. 2017 Apr 6;12(4):e0174805. doi: 10.1371/journal.pone.0174805 (PMC5383224; doi:10.1371/journal.pone.0174805)
Supplement: S2 Supporting information — SPSS full models and output. (DOC) [file pone.0174805.s006.doc]

MIXED HbA1C_Lgx1 BY Groep Tijd WITH Leeftijd Geslacht VLt3EU VLt3SU
  /CRITERIA=CIN(95) MXITER(100) MXSTEP(10) SCORING(1) SINGULAR(0.000000000001) HCONVERGE(0,
    ABSOLUTE) LCONVERGE(0, ABSOLUTE) PCONVERGE(0.000001, ABSOLUTE)
  /FIXED=Tijd Groep*Tijd Groep Leeftijd Geslacht VLt3EU VLt3SU | SSTYPE(3)
  /METHOD=REML
  /REPEATED=Tijd | SUBJECT(Volgnummer) COVTYPE(UN).


Mixed Model Analysis


Notes	
Output Created	09-FEB-2017 10:03:16	
Comments		
Input	Data	C:\Users\u0093379\Documents\Onderzoek\PhD\CM\Deelnemers BIBt1t3_long format.sav	
	Active Dataset	DataSet1	
	Filter	<none>	
	Weight	<none>	
	Split File	<none>	
	N of Rows in Working Data File	96	
Missing Value Handling	Definition of Missing	User-defined missing values are treated as missing.	
	Cases Used	Statistics are based on all cases with valid data for all variables in the model.	
Syntax	MIXED HbA1C_Lgx1 BY Groep Tijd WITH Leeftijd Geslacht VLt3EU VLt3SU
  /CRITERIA=CIN(95) MXITER(100) MXSTEP(10) SCORING(1) SINGULAR(0.000000000001) HCONVERGE(0,
    ABSOLUTE) LCONVERGE(0, ABSOLUTE) PCONVERGE(0.000001, ABSOLUTE)
  /FIXED=Tijd Groep*Tijd Groep Leeftijd Geslacht VLt3EU VLt3SU | SSTYPE(3)
  /METHOD=REML
  /REPEATED=Tijd | SUBJECT(Volgnummer) COVTYPE(UN).	
Resources	Processor Time	00:00:00,02	
	Elapsed Time	00:00:00,06	


[DataSet1] C:\Users\u0093379\Documents\Onderzoek\PhD\CM\Deelnemers BIBt1t3_long format.sav


Model Dimensiona	
	Number of Levels	Covariance Structure	Number of Parameters	Subject Variables	
Fixed Effects	Intercept	1		1		
	Tijd	2		1		
	Groep * Tijd	4		2		
	Groep	2		0		
	Leeftijd	1		1		
	Geslacht	1		1		
	VLt3EU	1		1		
	VLt3SU	1		1		
Repeated Effects	Tijd	2	Unstructured	3	Volgnummer	
Total	15		11		

Model Dimensiona	
	Number of Subjects	
Fixed Effects	Intercept		
	Tijd		
	Groep * Tijd		
	Groep		
	Leeftijd		
	Geslacht		
	VLt3EU		
	VLt3SU		
Repeated Effects	Tijd	39	
Total		

a. Dependent Variable: HbA1C_Lgx1.	


Information Criteriaa	
-2 Restricted Log Likelihood	-228,089	
Akaike's Information Criterion (AIC)	-222,089	
Hurvich and Tsai's Criterion (AICC)	-221,696	
Bozdogan's Criterion (CAIC)	-212,566	
Schwarz's Bayesian Criterion (BIC)	-215,566	

The information criteria are displayed in smaller-is-better form.a	
a. Dependent Variable: HbA1C_Lgx1.	


Fixed Effects


Type III Tests of Fixed Effectsa	
Source	Numerator df	Denominator df	F	Sig.	
Intercept	1	32,991	347,805	,000	
Tijd	1	32,727	1,481	,232	
Groep * Tijd	1	32,755	,427	,518	
Groep	1	32,855	,093	,762	
Leeftijd	1	32,944	2,225	,145	
Geslacht	1	32,881	1,610	,213	
VLt3EU	1	32,124	,804	,377	
VLt3SU	1	32,967	7,041	,012	

a. Dependent Variable: HbA1C_Lgx1.	


Covariance Parameters


Estimates of Covariance Parametersa	
Parameter	Estimate	Std. Error	
Repeated Measures	UN (1,1)	,001893	,000473	
	UN (2,1)	,001394	,000407	
	UN (2,2)	,001737	,000438	

a. Dependent Variable: HbA1C_Lgx1.	


MIXED HbA1C_Lgx1 BY Groep Tijd
  /CRITERIA=CIN(95) MXITER(100) MXSTEP(10) SCORING(1) SINGULAR(0.000000000001) HCONVERGE(0,
    ABSOLUTE) LCONVERGE(0, ABSOLUTE) PCONVERGE(0.000001, ABSOLUTE)
  /FIXED=Tijd Groep*Tijd Groep | SSTYPE(3)
  /METHOD=REML
  /REPEATED=Tijd | SUBJECT(Volgnummer) COVTYPE(UN).


Mixed Model Analysis


Notes	
Output Created	09-FEB-2017 10:03:16	
Comments		
Input	Data	C:\Users\u0093379\Documents\Onderzoek\PhD\CM\Deelnemers BIBt1t3_long format.sav	
	Active Dataset	DataSet1	
	Filter	<none>	
	Weight	<none>	
	Split File	<none>	
	N of Rows in Working Data File	96	
Missing Value Handling	Definition of Missing	User-defined missing values are treated as missing.	
	Cases Used	Statistics are based on all cases with valid data for all variables in the model.	
Syntax	MIXED HbA1C_Lgx1 BY Groep Tijd
  /CRITERIA=CIN(95) MXITER(100) MXSTEP(10) SCORING(1) SINGULAR(0.000000000001) HCONVERGE(0,
    ABSOLUTE) LCONVERGE(0, ABSOLUTE) PCONVERGE(0.000001, ABSOLUTE)
  /FIXED=Tijd Groep*Tijd Groep | SSTYPE(3)
  /METHOD=REML
  /REPEATED=Tijd | SUBJECT(Volgnummer) COVTYPE(UN).	
Resources	Processor Time	00:00:00,02	
	Elapsed Time	00:00:00,04	


Model Dimensiona	
	Number of Levels	Covariance Structure	Number of Parameters	Subject Variables	
Fixed Effects	Intercept	1		1		
	Tijd	2		1		
	Groep * Tijd	4		2		
	Groep	2		0		
Repeated Effects	Tijd	2	Unstructured	3	Volgnummer	
Total	11		7		

Model Dimensiona	
	Number of Subjects	
Fixed Effects	Intercept		
	Tijd		
	Groep * Tijd		
	Groep		
Repeated Effects	Tijd	48	
Total		

a. Dependent Variable: HbA1C_Lgx1.	


Information Criteriaa	
-2 Restricted Log Likelihood	-290,239	
Akaike's Information Criterion (AIC)	-284,239	
Hurvich and Tsai's Criterion (AICC)	-283,923	
Bozdogan's Criterion (CAIC)	-274,093	
Schwarz's Bayesian Criterion (BIC)	-277,093	

The information criteria are displayed in smaller-is-better form.a	
a. Dependent Variable: HbA1C_Lgx1.	


Fixed Effects


Type III Tests of Fixed Effectsa	
Source	Numerator df	Denominator df	F	Sig.	
Intercept	1	45,650	20858,490	,000	
Tijd	1	36,197	,561	,459	
Groep * Tijd	1	36,197	,534	,470	
Groep	1	45,650	,059	,809	

a. Dependent Variable: HbA1C_Lgx1.	


Covariance Parameters


Estimates of Covariance Parametersa	
Parameter	Estimate	Std. Error	
Repeated Measures	UN (1,1)	,002009	,000423	
	UN (2,1)	,001486	,000374	
	UN (2,2)	,001871	,000425	

a. Dependent Variable: HbA1C_Lgx1.	

MIXED ZMWTafstand BY Groep Tijd WITH Geslacht Leeftijd BMIbaseline VerschilBMIt3t1
  /CRITERIA=CIN(95) MXITER(100) MXSTEP(10) SCORING(1) SINGULAR(0.000000000001) HCONVERGE(0,
    ABSOLUTE) LCONVERGE(0, ABSOLUTE) PCONVERGE(0.000001, ABSOLUTE)
  /FIXED=Groep Tijd Geslacht Leeftijd BMIbaseline VerschilBMIt3t1 Groep*Tijd | SSTYPE(3)
  /METHOD=REML
  /REPEATED=Tijd | SUBJECT(Volgnummer) COVTYPE(UN).


Mixed Model Analysis


Notes	
Output Created	09-FEB-2017 10:11:10	
Comments		
Input	Data	C:\Users\u0093379\Documents\Onderzoek\PhD\CM\Deelnemers BIBt1t3_long format.sav	
	Active Dataset	DataSet1	
	Filter	<none>	
	Weight	<none>	
	Split File	<none>	
	N of Rows in Working Data File	96	
Missing Value Handling	Definition of Missing	User-defined missing values are treated as missing.	
	Cases Used	Statistics are based on all cases with valid data for all variables in the model.	
Syntax	MIXED ZMWTafstand BY Groep Tijd WITH Geslacht Leeftijd BMIbaseline VerschilBMIt3t1
  /CRITERIA=CIN(95) MXITER(100) MXSTEP(10) SCORING(1) SINGULAR(0.000000000001) HCONVERGE(0,
    ABSOLUTE) LCONVERGE(0, ABSOLUTE) PCONVERGE(0.000001, ABSOLUTE)
  /FIXED=Groep Tijd Geslacht Leeftijd BMIbaseline VerschilBMIt3t1 Groep*Tijd | SSTYPE(3)
  /METHOD=REML
  /REPEATED=Tijd | SUBJECT(Volgnummer) COVTYPE(UN).	
Resources	Processor Time	00:00:00,02	
	Elapsed Time	00:00:00,05	


Model Dimensiona	
	Number of Levels	Covariance Structure	Number of Parameters	
Fixed Effects	Intercept	1		1	
	Groep	2		1	
	Tijd	2		1	
	Geslacht	1		1	
	Leeftijd	1		1	
	BMIbaseline	1		1	
	VerschilBMIt3t1	1		1	
	Groep * Tijd	4		1	
Repeated Effects	Tijd	2	Unstructured	3	
Total	15		11	

Model Dimensiona	
	Subject Variables	Number of Subjects	
Fixed Effects	Intercept			
	Groep			
	Tijd			
	Geslacht			
	Leeftijd			
	BMIbaseline			
	VerschilBMIt3t1			
	Groep * Tijd			
Repeated Effects	Tijd	Volgnummer	40	
Total			

a. Dependent Variable: ZMWTafstand.	


Information Criteriaa	
-2 Restricted Log Likelihood	761,733	
Akaike's Information Criterion (AIC)	767,733	
Hurvich and Tsai's Criterion (AICC)	768,091	
Bozdogan's Criterion (CAIC)	777,521	
Schwarz's Bayesian Criterion (BIC)	774,521	

The information criteria are displayed in smaller-is-better form.a	
a. Dependent Variable: ZMWTafstand.	


Fixed Effects


Type III Tests of Fixed Effectsa	
Source	Numerator df	Denominator df	F	Sig.	
Intercept	1	34,128	82,277	,000	
Groep	1	34,152	,001	,972	
Tijd	1	37,270	5,400	,026	
Geslacht	1	34,098	5,862	,021	
Leeftijd	1	33,995	23,810	,000	
BMIbaseline	1	34,109	,873	,357	
VerschilBMIt3t1	1	34,012	1,102	,301	
Groep * Tijd	1	37,270	1,773	,191	

a. Dependent Variable: ZMWTafstand.	


Covariance Parameters


Estimates of Covariance Parametersa	
Parameter	Estimate	Std. Error	
Repeated Measures	UN (1,1)	4806,438072	1162,733848	
	UN (2,1)	4302,959941	1086,931029	
	UN (2,2)	4509,135277	1093,568992	

a. Dependent Variable: ZMWTafstand.	


MIXED ZMWTafstand BY Groep Tijd
  /CRITERIA=CIN(95) MXITER(100) MXSTEP(10) SCORING(1) SINGULAR(0.000000000001) HCONVERGE(0,
    ABSOLUTE) LCONVERGE(0, ABSOLUTE) PCONVERGE(0.000001, ABSOLUTE)
  /FIXED=Groep Tijd Groep*Tijd | SSTYPE(3)
  /METHOD=REML
  /REPEATED=Tijd | SUBJECT(Volgnummer) COVTYPE(UN).


Mixed Model Analysis


Notes	
Output Created	09-FEB-2017 10:11:10	
Comments		
Input	Data	C:\Users\u0093379\Documents\Onderzoek\PhD\CM\Deelnemers BIBt1t3_long format.sav	
	Active Dataset	DataSet1	
	Filter	<none>	
	Weight	<none>	
	Split File	<none>	
	N of Rows in Working Data File	96	
Missing Value Handling	Definition of Missing	User-defined missing values are treated as missing.	
	Cases Used	Statistics are based on all cases with valid data for all variables in the model.	
Syntax	MIXED ZMWTafstand BY Groep Tijd
  /CRITERIA=CIN(95) MXITER(100) MXSTEP(10) SCORING(1) SINGULAR(0.000000000001) HCONVERGE(0,
    ABSOLUTE) LCONVERGE(0, ABSOLUTE) PCONVERGE(0.000001, ABSOLUTE)
  /FIXED=Groep Tijd Groep*Tijd | SSTYPE(3)
  /METHOD=REML
  /REPEATED=Tijd | SUBJECT(Volgnummer) COVTYPE(UN).	
Resources	Processor Time	00:00:00,00	
	Elapsed Time	00:00:00,04	


Model Dimensiona	
	Number of Levels	Covariance Structure	Number of Parameters	Subject Variables	
Fixed Effects	Intercept	1		1		
	Groep	2		1		
	Tijd	2		1		
	Groep * Tijd	4		1		
Repeated Effects	Tijd	2	Unstructured	3	Volgnummer	
Total	11		7		

Model Dimensiona	
	Number of Subjects	
Fixed Effects	Intercept		
	Groep		
	Tijd		
	Groep * Tijd		
Repeated Effects	Tijd	43	
Total		

a. Dependent Variable: ZMWTafstand.	


Information Criteriaa	
-2 Restricted Log Likelihood	839,199	
Akaike's Information Criterion (AIC)	845,199	
Hurvich and Tsai's Criterion (AICC)	845,523	
Bozdogan's Criterion (CAIC)	855,269	
Schwarz's Bayesian Criterion (BIC)	852,269	

The information criteria are displayed in smaller-is-better form.a	
a. Dependent Variable: ZMWTafstand.	


Fixed Effects


Type III Tests of Fixed Effectsa	
Source	Numerator df	Denominator df	F	Sig.	
Intercept	1	41,197	1762,610	,000	
Groep	1	41,197	,124	,727	
Tijd	1	37,236	5,388	,026	
Groep * Tijd	1	37,236	1,738	,195	

a. Dependent Variable: ZMWTafstand.	


Covariance Parameters


Estimates of Covariance Parametersa	
Parameter	Estimate	Std. Error	
Repeated Measures	UN (1,1)	7110,909926	1570,537270	
	UN (2,1)	6795,396278	1540,726134	
	UN (2,2)	7189,413047	1589,425622	

a. Dependent Variable: ZMWTafstand.	


MIXED PAtotaal_Lgx1 BY Groep Tijd WITH Geslacht Leeftijd BMIbaseline VerschilBMIt3t1 Daglengtet3 Temperatuurt3 Neerslagt3
  /CRITERIA=CIN(95) MXITER(100) MXSTEP(10) SCORING(1) SINGULAR(0.000000000001) HCONVERGE(0,
    ABSOLUTE) LCONVERGE(0, ABSOLUTE) PCONVERGE(0.000001, ABSOLUTE)
  /FIXED=Groep Tijd Geslacht Leeftijd BMIbaseline VerschilBMIt3t1 Daglengtet3 Temperatuurt3 Neerslagt3 Groep*Tijd | SSTYPE(3)
  /METHOD=REML
  /REPEATED=Tijd | SUBJECT(Volgnummer) COVTYPE(UN).


Mixed Model Analysis


Notes	
Output Created	09-FEB-2017 10:11:10	
Comments		
Input	Data	C:\Users\u0093379\Documents\Onderzoek\PhD\CM\Deelnemers BIBt1t3_long format.sav	
	Active Dataset	DataSet1	
	Filter	<none>	
	Weight	<none>	
	Split File	<none>	
	N of Rows in Working Data File	96	
Missing Value Handling	Definition of Missing	User-defined missing values are treated as missing.	
	Cases Used	Statistics are based on all cases with valid data for all variables in the model.	
Syntax	MIXED PAtotaal_Lgx1 BY Groep Tijd WITH Geslacht Leeftijd BMIbaseline VerschilBMIt3t1 Daglengtet3 Temperatuurt3 Neerslagt3
  /CRITERIA=CIN(95) MXITER(100) MXSTEP(10) SCORING(1) SINGULAR(0.000000000001) HCONVERGE(0,
    ABSOLUTE) LCONVERGE(0, ABSOLUTE) PCONVERGE(0.000001, ABSOLUTE)
  /FIXED=Groep Tijd Geslacht Leeftijd BMIbaseline VerschilBMIt3t1 Daglengtet3 Temperatuurt3 Neerslagt3 Groep*Tijd | SSTYPE(3)
  /METHOD=REML
  /REPEATED=Tijd | SUBJECT(Volgnummer) COVTYPE(UN).	
Resources	Processor Time	00:00:00,03	
	Elapsed Time	00:00:00,05	


Model Dimensiona	
	Number of Levels	Covariance Structure	Number of Parameters	
Fixed Effects	Intercept	1		1	
	Groep	2		1	
	Tijd	2		1	
	Geslacht	1		1	
	Leeftijd	1		1	
	BMIbaseline	1		1	
	VerschilBMIt3t1	1		1	
	Daglengtet3	1		1	
	Temperatuurt3	1		1	
	Neerslagt3	1		1	
	Groep * Tijd	4		1	
Repeated Effects	Tijd	2	Unstructured	3	
Total	18		14	

Model Dimensiona	
	Subject Variables	Number of Subjects	
Fixed Effects	Intercept			
	Groep			
	Tijd			
	Geslacht			
	Leeftijd			
	BMIbaseline			
	VerschilBMIt3t1			
	Daglengtet3			
	Temperatuurt3			
	Neerslagt3			
	Groep * Tijd			
Repeated Effects	Tijd	Volgnummer	36	
Total			

a. Dependent Variable: PAtotaal_Lgx1.	


Information Criteriaa	
-2 Restricted Log Likelihood	61,561	
Akaike's Information Criterion (AIC)	67,561	
Hurvich and Tsai's Criterion (AICC)	67,989	
Bozdogan's Criterion (CAIC)	76,844	
Schwarz's Bayesian Criterion (BIC)	73,844	

The information criteria are displayed in smaller-is-better form.a	
a. Dependent Variable: PAtotaal_Lgx1.	


Fixed Effects


Type III Tests of Fixed Effectsa	
Source	Numerator df	Denominator df	F	Sig.	
Intercept	1	26,908	,261	,614	
Groep	1	26,955	1,004	,325	
Tijd	1	33,456	5,664	,023	
Geslacht	1	26,873	,095	,760	
Leeftijd	1	26,887	1,310	,263	
BMIbaseline	1	27,023	,046	,832	
VerschilBMIt3t1	1	26,891	,495	,488	
Daglengtet3	1	26,980	,959	,336	
Temperatuurt3	1	27,012	,117	,735	
Neerslagt3	1	28,015	,013	,910	
Groep * Tijd	1	33,456	1,259	,270	

a. Dependent Variable: PAtotaal_Lgx1.	


Covariance Parameters


Estimates of Covariance Parametersa	
Parameter	Estimate	Std. Error	
Repeated Measures	UN (1,1)	,087505	,024242	
	UN (2,1)	,052914	,021166	
	UN (2,2)	,106248	,030094	

a. Dependent Variable: PAtotaal_Lgx1.	


MIXED PAtotaal_Lgx1 BY Groep Tijd
  /CRITERIA=CIN(95) MXITER(100) MXSTEP(10) SCORING(1) SINGULAR(0.000000000001) HCONVERGE(0,
    ABSOLUTE) LCONVERGE(0, ABSOLUTE) PCONVERGE(0.000001, ABSOLUTE)
  /FIXED=Groep Tijd Groep*Tijd | SSTYPE(3)
  /METHOD=REML
  /REPEATED=Tijd | SUBJECT(Volgnummer) COVTYPE(UN).


Mixed Model Analysis


Notes	
Output Created	09-FEB-2017 10:11:11	
Comments		
Input	Data	C:\Users\u0093379\Documents\Onderzoek\PhD\CM\Deelnemers BIBt1t3_long format.sav	
	Active Dataset	DataSet1	
	Filter	<none>	
	Weight	<none>	
	Split File	<none>	
	N of Rows in Working Data File	96	
Missing Value Handling	Definition of Missing	User-defined missing values are treated as missing.	
	Cases Used	Statistics are based on all cases with valid data for all variables in the model.	
Syntax	MIXED PAtotaal_Lgx1 BY Groep Tijd
  /CRITERIA=CIN(95) MXITER(100) MXSTEP(10) SCORING(1) SINGULAR(0.000000000001) HCONVERGE(0,
    ABSOLUTE) LCONVERGE(0, ABSOLUTE) PCONVERGE(0.000001, ABSOLUTE)
  /FIXED=Groep Tijd Groep*Tijd | SSTYPE(3)
  /METHOD=REML
  /REPEATED=Tijd | SUBJECT(Volgnummer) COVTYPE(UN).	
Resources	Processor Time	00:00:00,02	
	Elapsed Time	00:00:00,04	


Model Dimensiona	
	Number of Levels	Covariance Structure	Number of Parameters	Subject Variables	
Fixed Effects	Intercept	1		1		
	Groep	2		1		
	Tijd	2		1		
	Groep * Tijd	4		1		
Repeated Effects	Tijd	2	Unstructured	3	Volgnummer	
Total	11		7		

Model Dimensiona	
	Number of Subjects	
Fixed Effects	Intercept		
	Groep		
	Tijd		
	Groep * Tijd		
Repeated Effects	Tijd	46	
Total		

a. Dependent Variable: PAtotaal_Lgx1.	


Information Criteriaa	
-2 Restricted Log Likelihood	54,841	
Akaike's Information Criterion (AIC)	60,841	
Hurvich and Tsai's Criterion (AICC)	61,152	
Bozdogan's Criterion (CAIC)	71,024	
Schwarz's Bayesian Criterion (BIC)	68,024	

The information criteria are displayed in smaller-is-better form.a	
a. Dependent Variable: PAtotaal_Lgx1.	


Fixed Effects


Type III Tests of Fixed Effectsa	
Source	Numerator df	Denominator df	F	Sig.	
Intercept	1	40,176	1199,217	,000	
Groep	1	40,176	,000	,999	
Tijd	1	38,290	4,233	,047	
Groep * Tijd	1	38,290	,323	,573	

a. Dependent Variable: PAtotaal_Lgx1.	


Covariance Parameters


Estimates of Covariance Parametersa	
Parameter	Estimate	Std. Error	
Repeated Measures	UN (1,1)	,099349	,021362	
	UN (2,1)	,042327	,019772	
	UN (2,2)	,116500	,027379	

a. Dependent Variable: PAtotaal_Lgx1.	


MIXED SWTPA BY Groep Tijd WITH Geslacht Leeftijd BMIbaseline VerschilBMIt3t1 Daglengtet3 Temperatuurt3 Neerslagt3
  /CRITERIA=CIN(95) MXITER(100) MXSTEP(10) SCORING(1) SINGULAR(0.000000000001) HCONVERGE(0,
    ABSOLUTE) LCONVERGE(0, ABSOLUTE) PCONVERGE(0.000001, ABSOLUTE)
  /FIXED=Groep Tijd Geslacht Leeftijd BMIbaseline VerschilBMIt3t1 Daglengtet3 Temperatuurt3 Neerslagt3 Groep*Tijd | SSTYPE(3)
  /METHOD=REML
  /REPEATED=Tijd | SUBJECT(Volgnummer) COVTYPE(UN).


Mixed Model Analysis


Notes	
Output Created	09-FEB-2017 10:11:11	
Comments		
Input	Data	C:\Users\u0093379\Documents\Onderzoek\PhD\CM\Deelnemers BIBt1t3_long format.sav	
	Active Dataset	DataSet1	
	Filter	<none>	
	Weight	<none>	
	Split File	<none>	
	N of Rows in Working Data File	96	
Missing Value Handling	Definition of Missing	User-defined missing values are treated as missing.	
	Cases Used	Statistics are based on all cases with valid data for all variables in the model.	
Syntax	MIXED SWTPA BY Groep Tijd WITH Geslacht Leeftijd BMIbaseline VerschilBMIt3t1 Daglengtet3 Temperatuurt3 Neerslagt3
  /CRITERIA=CIN(95) MXITER(100) MXSTEP(10) SCORING(1) SINGULAR(0.000000000001) HCONVERGE(0,
    ABSOLUTE) LCONVERGE(0, ABSOLUTE) PCONVERGE(0.000001, ABSOLUTE)
  /FIXED=Groep Tijd Geslacht Leeftijd BMIbaseline VerschilBMIt3t1 Daglengtet3 Temperatuurt3 Neerslagt3 Groep*Tijd | SSTYPE(3)
  /METHOD=REML
  /REPEATED=Tijd | SUBJECT(Volgnummer) COVTYPE(UN).	
Resources	Processor Time	00:00:00,02	
	Elapsed Time	00:00:00,07	


Model Dimensiona	
	Number of Levels	Covariance Structure	Number of Parameters	
Fixed Effects	Intercept	1		1	
	Groep	2		1	
	Tijd	2		1	
	Geslacht	1		1	
	Leeftijd	1		1	
	BMIbaseline	1		1	
	VerschilBMIt3t1	1		1	
	Daglengtet3	1		1	
	Temperatuurt3	1		1	
	Neerslagt3	1		1	
	Groep * Tijd	4		1	
Repeated Effects	Tijd	2	Unstructured	3	
Total	18		14	

Model Dimensiona	
	Subject Variables	Number of Subjects	
Fixed Effects	Intercept			
	Groep			
	Tijd			
	Geslacht			
	Leeftijd			
	BMIbaseline			
	VerschilBMIt3t1			
	Daglengtet3			
	Temperatuurt3			
	Neerslagt3			
	Groep * Tijd			
Repeated Effects	Tijd	Volgnummer	36	
Total			

a. Dependent Variable: SWTPA.	


Information Criteriaa	
-2 Restricted Log Likelihood	653,367	
Akaike's Information Criterion (AIC)	659,367	
Hurvich and Tsai's Criterion (AICC)	659,838	
Bozdogan's Criterion (CAIC)	668,389	
Schwarz's Bayesian Criterion (BIC)	665,389	

The information criteria are displayed in smaller-is-better form.a	
a. Dependent Variable: SWTPA.	


Fixed Effects


Type III Tests of Fixed Effectsa	
Source	Numerator df	Denominator df	F	Sig.	
Intercept	1	24,803	10,267	,004	
Groep	1	24,538	,697	,412	
Tijd	1	28,017	,527	,474	
Geslacht	1	25,695	2,776	,108	
Leeftijd	1	24,491	7,394	,012	
BMIbaseline	1	24,448	,259	,616	
VerschilBMIt3t1	1	25,452	,425	,520	
Daglengtet3	1	25,824	1,043	,317	
Temperatuurt3	1	25,481	,209	,651	
Neerslagt3	1	26,510	3,494	,073	
Groep * Tijd	1	28,226	,222	,641	

a. Dependent Variable: SWTPA.	


Covariance Parameters


Estimates of Covariance Parametersa	
Parameter	Estimate	Std. Error	
Repeated Measures	UN (1,1)	5471,494554	1554,420688	
	UN (2,1)	3088,524185	1225,528740	
	UN (2,2)	4506,080272	1287,730585	

a. Dependent Variable: SWTPA.	


MIXED SWTPA BY Groep Tijd
  /CRITERIA=CIN(95) MXITER(100) MXSTEP(10) SCORING(1) SINGULAR(0.000000000001) HCONVERGE(0,
    ABSOLUTE) LCONVERGE(0, ABSOLUTE) PCONVERGE(0.000001, ABSOLUTE)
  /FIXED=Groep Tijd Groep*Tijd | SSTYPE(3)
  /METHOD=REML
  /REPEATED=Tijd | SUBJECT(Volgnummer) COVTYPE(UN).


Mixed Model Analysis


Notes	
Output Created	09-FEB-2017 10:11:11	
Comments		
Input	Data	C:\Users\u0093379\Documents\Onderzoek\PhD\CM\Deelnemers BIBt1t3_long format.sav	
	Active Dataset	DataSet1	
	Filter	<none>	
	Weight	<none>	
	Split File	<none>	
	N of Rows in Working Data File	96	
Missing Value Handling	Definition of Missing	User-defined missing values are treated as missing.	
	Cases Used	Statistics are based on all cases with valid data for all variables in the model.	
Syntax	MIXED SWTPA BY Groep Tijd
  /CRITERIA=CIN(95) MXITER(100) MXSTEP(10) SCORING(1) SINGULAR(0.000000000001) HCONVERGE(0,
    ABSOLUTE) LCONVERGE(0, ABSOLUTE) PCONVERGE(0.000001, ABSOLUTE)
  /FIXED=Groep Tijd Groep*Tijd | SSTYPE(3)
  /METHOD=REML
  /REPEATED=Tijd | SUBJECT(Volgnummer) COVTYPE(UN).	
Resources	Processor Time	00:00:00,02	
	Elapsed Time	00:00:00,05	


Model Dimensiona	
	Number of Levels	Covariance Structure	Number of Parameters	Subject Variables	
Fixed Effects	Intercept	1		1		
	Groep	2		1		
	Tijd	2		1		
	Groep * Tijd	4		1		
Repeated Effects	Tijd	2	Unstructured	3	Volgnummer	
Total	11		7		

Model Dimensiona	
	Number of Subjects	
Fixed Effects	Intercept		
	Groep		
	Tijd		
	Groep * Tijd		
Repeated Effects	Tijd	46	
Total		

a. Dependent Variable: SWTPA.	


Information Criteriaa	
-2 Restricted Log Likelihood	825,056	
Akaike's Information Criterion (AIC)	831,056	
Hurvich and Tsai's Criterion (AICC)	831,409	
Bozdogan's Criterion (CAIC)	840,886	
Schwarz's Bayesian Criterion (BIC)	837,886	

The information criteria are displayed in smaller-is-better form.a	
a. Dependent Variable: SWTPA.	


Fixed Effects


Type III Tests of Fixed Effectsa	
Source	Numerator df	Denominator df	F	Sig.	
Intercept	1	43,913	113,815	,000	
Groep	1	43,913	,284	,597	
Tijd	1	30,513	,088	,769	
Groep * Tijd	1	30,513	,032	,859	

a. Dependent Variable: SWTPA.	


Covariance Parameters


Estimates of Covariance Parametersa	
Parameter	Estimate	Std. Error	
Repeated Measures	UN (1,1)	6340,619800	1402,594145	
	UN (2,1)	4195,326343	1208,536748	
	UN (2,2)	5763,402654	1413,324084	

a. Dependent Variable: SWTPA.	
